# Duplication and Gene Conversion in the *Drosophila melanogaster* Genome

**DOI:** 10.1371/journal.pgen.1000305

**Published:** 2008-12-12

**Authors:** Naoki Osada, Hideki Innan

**Affiliations:** 1 National Institute of Biomedical Innovation, Osaka, Japan; 2 Graduate University for Advanced Studies, Hayama, Japan; University of Aarhus, Denmark

## Abstract

Using the genomic sequences of *Drosophila melanogaster* subgroup, the pattern of gene duplications was investigated with special attention to interlocus gene conversion. Our fine-scale analysis with careful visual inspections enabled accurate identification of a number of duplicated blocks (genomic regions). The orthologous parts of those duplicated blocks were also identified in the *D. simulans* and *D. sechellia* genomes, by which we were able to clearly classify the duplicated blocks into post- and pre-speciation blocks. We found 31 post-speciation duplicated genes, from which the rate of gene duplication (from one copy to two copies) is estimated to be 1.0×10^−9^ per single-copy gene per year. The role of interlocus gene conversion was observed in several respects in our data: (1) synonymous divergence between a duplicated pair is overall very low. Consequently, the gene duplication rate would be seriously overestimated by counting duplicated genes with low divergence; (2) the sizes of young duplicated blocks are generally large. We postulate that the degeneration of gene conversion around the edges could explain the shrinkage of “identifiable” duplicated regions; and (3) elevated paralogous divergence is observed around the edges in many duplicated blocks, supporting our gene conversion–degeneration model. Our analysis demonstrated that gene conversion between duplicated regions is a common and genome-wide phenomenon in the *Drosophila* genomes, and that its role should be especially significant in the early stages of duplicated genes. Based on a population genetic prediction, we applied a new genome-scan method to test for signatures of selection for neofunctionalization and found a strong signature in a pair of transporter genes.

## Author Summary

Eukaryote genomes have a number of duplicated genes, which could potentially coevolve by exchanging DNA sequences by interlocus gene conversion. However, the extent of gene conversion on a genomic scale is not well understood, except that an extensive role of gene conversion was reported in yeast. Here, we show a second evaluation of the role of gene conversion by analyzing multiple genomes in the *D. melanogaster* subgroup. We found that most of young duplicated genes have experienced gene conversion, although not as extensively as yeast. We further performed fine-scale analysis of duplicated DNA sequences and estimated the gene duplication rate. Our estimate turned out to be much smaller than that of a commonly used method, which usually causes an overestimation when gene conversion is active. The role of positive selection for neofunctionalization was inferred by applying a novel test. Our results suggest that interlocus gene conversion could be a crucial mutational mechanism in the evolution of duplicated genes in eukaryote genomes and that the effect of gene conversion should be taken into account when analyzing molecular evolution of duplicated genes.

## Introduction

As proposed almost four decades ago, gene duplication is one of the major sources to create genetic novelty [1]. Gene duplication followed by the fixation of a mutation providing a slightly different function should be a possible scenario of the evolution of new gene function via duplication (*i.e.*, neofunctionalization of a duplicated gene). To understand the contribution of this mechanism to genomic evolution, we need to answer at least two fundamental questions: “How often does gene duplication occur?” and “What are the signatures of natural selection operating on a mutation providing neofunctionalization?”

Using the Drosophila genomes as a model, this article addresses these two questions with special attention to gene conversion between duplicated genes. Gene conversion is one outcome of a recombination event, which is usually modeled as a copy-and-paste event [2],[3]. Interlocus gene conversion transfers a DNA fragment in one region to the corresponding place in another paralogous region; subsequently, the transferred region becomes identical. With frequent gene conversion, the paralogous regions keep their sequences very similar for a long time, resulting in the well-known phenomenon of concerted evolution [4],[5],[6],[7]. Although concerted evolution was first demonstrated more than 30 years ago [8], its genomic impact has been unveiled only recently. It is increasingly recognized that interlocus gene conversion can be a genome-wide phenomenon in a wide range of organisms from yeast to higher eukaryotes [9],[10],[11],[12], although the extent depends on species.

There are strong reasons why it is important to elucidate the role of gene conversion after gene duplication in order to address the above two questions. A simple ad-hoc method of estimating the gene duplication rate is to count gene-pairs of low divergence (presumably young) in the genome [13]. This method works only when the nucleotide divergence between the duplicated genes follows the molecular clock [14], in which case gene pairs with low divergence are indeed young. However, Teshima and Innan [15] theoretically demonstrated that this method will cause a serious overestimation of the gene duplication rate when a number of duplicated genes undergo concerted evolution and Gao and Innan [11] showed that this is the case for the yeast genome (*Saccharomyces cerevisiae*). In such a situation, because the divergence between duplicated genes does not necessarily reflect their ages, other methods should be used. In the study of Gao and Innan [11], a comparative genomic approach was used, in which genomic sequences of several closely related species of *S. cerevisiae* [16],[17] were involved. The gene duplication rate was estimated by directly mapping duplication events on a phylogeny of those species, which was two orders of magnitude lower than the divergence-based estimate.

Now, recent genome sequence data of *Drosophila* [18] provide the second opportunity to evaluate the role of interlocus gene conversion in eukaryotes by using comparative genomic approaches. Such followup studies are important to examine the generality of the conclusion obtained from yeasts [11]. The situation of the *Drosophila* genome data is similar to that of yeast. There is a completed genome sequence data available for a model species (*D. melanogaster* in fruit flies and *S. cerevisiae* in yeasts), and its relatives' genomes are sequenced at various levels in quantity and quality. Therefore, in our comparative genomic study, the finished *D. melanogaster* genome [19] plays the key role, as well as in other studies [*e.g.*],[18],^20^,^21,22^. In other words, the *D. melanogaster* genome serves as a reliable template to understand the genomic organization of the other species, especially when most of the 11 newly sequenced genomes are not yet assembled into chromosomes (exceptions are *D. simulans* and *D. yakuba*) [19].

Gene duplications in *Drosophila* have been extensively studied in various scales by using the comparative genomic data [18]. For example, Hahn et al. [22] investigated the pattern of gene duplication and loss in gene families that are defined as groups of homologous genes. Some gene families consist of hundreds of copy members. Based on the changes in the copy number along evolutionary history, the rates of duplication and loss were estimated. Heger and Ponting [21] also performed comprehensive evolutionary analysis of homologous genes across the 12 species and found an excess of low-divergence duplicated genes in the terminal branches of the 12-species tree, which was in agreement with the observation of Lynch and Conery [13]. However, in those long-term evolutionary analyses, it was very difficult to elucidate the role of gene conversion because it plays significant roles in early stages of duplicated genes.

This article primarily focuses on the patterns of nucleotide evolution in relatively young duplicates, where gene conversion is likely to be active. We restrict our analysis to duplication events, by which single-copy genes become two-copy duplicated genes (1→2 duplication) to exclude ambiguity caused by multiple complex duplications in large multigene families. While some large families exhibit evidence for expansion in size and rapid amino acid changes [22], the molecular evolution of two-copy duplicates is relatively slow. This makes it possible to trace the history of duplicates at the DNA level in the *D. melanogaster* subgroup, from which we performed a fine-scale analysis of the duplicated genomic regions including non-coding regions. We were able to identify what part of the genome was duplicated in *D. melanogaster* and *D. simulans*, from which we inferred when the duplication event occurred (*i.e.*, whether it was before or after the speciation of the two species). With these data, we demonstrated a significant role of gene conversion between young duplicated genes, and obtained an estimate of the gene duplication rate, which is much lower than that of the divergence-based method used by Lynch and Conery [13].

The comparative genomic data are also used to detect the signatures of natural selection for neofunctionalization. The neofunctionalization process can be initiated by a single beneficial mutation, which provides a slightly different function so that selection works to maintain this mutation. However, it is usually very difficult to detect the signature of selection in DNA sequence data, unless a number of nonsynonymous nucleotide substitutions occur at a faster rate than synonymous substitutions [*e.g.*,23]. Recently, Teshima and Innan [24] proposed a novel idea to detect signatures of neofunctionalization, which works best when the duplicated regions are undergoing concerted evolution. When there is gene conversion between duplicated genes, a newly arisen neofunctionalized mutation could be erased by gene conversion. Therefore, the neofunctionalized mutation can be stably maintained in the population only when its selective advantage is much larger than the rate of gene conversion [25]. Under these conditions, deleterious (at least less beneficial) gene conversion is immediately eliminated from the population. Teshima and Innan [24] found that the maintenance of a neofunctionalized mutation through the balance of strong selection and gene conversion continues for a relatively long time. In this period, a local peak of the divergence between the duplicates emerges because of the lack of paralogous DNA exchanges in this region. This high level of divergence accumulated around the site of the neofunctionalized mutation is contrasted with low divergence in regions away from the site. Therefore, Teshima and Innan [24] suggested the possibility of using this signature of selection in a genome scan for recent neofunctionalization. The idea was applied to our data, and we found a strong signature of recent neofunctionalization in a pair of transporter genes.

## Results

**Figure 1:**
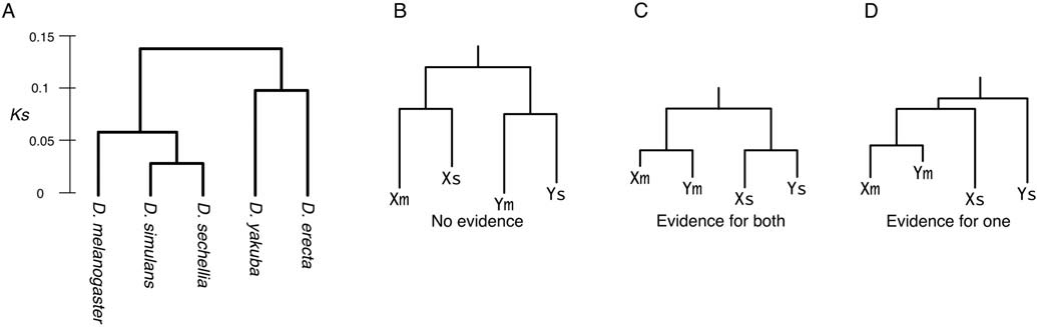
(A) Phylogenetic relationship of the five species in the *D. melanogaster* subgroup. The distance is based on the nucleotide divergence at synonymous sites (*K_S_*). Modified from Figure 2B of [21]. (B–D) Evidence for gene conversion in the gene tree shapes. Xm and Ym represent a pair of duplicated gene in *D. melanogaster*, and their orthologs in *D. simulans* (or in *D. sechellia*) are denoted by Xs and Ys. See text for details.

Overall, our basic strategy is that duplicated regions are identified in the *D. melanogaster* genome by taking advantage of its data quality. The genome is sequenced with high depth [19] and coding genes are well annotated [26]. Then, using those data as templates, we trace their evolutionary histories of the other four sequenced species in the *D. melanogaster* subgroup (*D. simulans*, *D. sechellia*, *D. yakuba*, and *D. erecta*). A species tree of the subgroup is shown in Figure 1A. In practice, we first identified two-copy duplicated genes in the *D. melanogaster* genome, and their orthologous regions were identified in their relatives' genomes. The rate of success depends on the evolutionary distance from *D. melanogaster* and the coverage of genomic sequences. To look for evidence for presence of the duplicated regions identified in *D. melanogaster*, we used the assembly of *D. simulans* and *D. sechellia*. For *D. simulans*, seven strains in total are sequenced at different coverage: roughly 4-fold whole genome shotgun (WGS) sequence data are available for the w501 strain and the WGS coverage is about 1× for the other six strains. The assembly of *D. simulans* consists of the assembly of the w501 strain, in which gaps are filled with the assemblies from the other six strains. *D. sechellia* is very closely related with *D. simulans* (Figure 1A), and the WGS coverage of the genomic sequence of *D. sechellia* is about 4-fold. The identification of duplicated genomic regions were quite successful for these two species.

We also extended this analysis to the next closest relatives, *D. yakuba* and *D. erecta*. However, we found it quite difficult to fully align their sequences with *D. melanogaster* in non-coding regions. In most cases, our strategy worked only partially for non-coding regions, making it difficult to determine the orthology. Therefore, we used those partially identified regions as outgroups in the analysis. *D. yakuba* is mainly used for this purpose because its genome is assembled into chromosomes. When we found multiple homologous copies, the best aligned one was used as an outgroup. It seems that the upper limit of comparative analysis of non-coding regions might be within the *D. melanogaster* subgroup in the 12 sequenced *Drosophila* species.

### Pattern of Gene Duplications and Gene Conversion

Sixty three pairs of two-copy duplicated genes with synonymous divergence *K_S_*<0.2 were identified in the *D. melanogaster* genome (see Methods). This *K_S_* cutoff value was chosen such that almost all duplicated genes in the *D. melanogaster* genome that appeared after the speciation of *D. melanogaster* and *D. simulans* can be detected. Note that the average *K_S_* between the two species is around 0.12 [21], so that the probability that *K_S_* between duplicates exceeds 0.2 should be very low. Then, the locations of these genes on the *D. melanogaster* genomic sequence were visually examined, and by using the BLASTN algorithm we identified duplicated genomic regions (blocks) that encompass the identified duplicated genes. It was found that the 63 duplicated genes belong to 55 duplication blocks: some of them are next to each other and belong to the same duplication blocks (summarized in Tables 1 and 2). Almost all duplicates are located on the same chromosomes. For each pair of the duplicated blocks, the one that is close to the telomere of the left arm of the chromosome was assigned to Xm and the other was assigned to Ym. These results are consistent with those of Fiston-Lavier et al. [27].

**Table 1:** Summary of the Post-speciation Duplicated Blocks.

Block ID	*D. melanogaster*		*D. simulans* (*D. sechellia*)						
	Region X (Xm)	Region Y (Ym)	Ortholog	*Ka*	*Ks*	(*s_X_*, *s_Y_*)	*L*	*I*	ancestral
Post1	3L:18462082-18462933	3L:18464853-18465699	3L:17797868-17798751	0.0000	0.0000	(1,1)	849.5	1919	NA
Post2	2L:15653163-15686759	2L:15686760-15721756	2L:15406396-15436548	0.0000	0.0000	(3,3)	34297	0	NA
Post3	2R:2882731-2889019	2R:2889020-2895307	2R:1689838-1695893	0.0000	0.0000	(2,3)	6288.5	0	NA
Post4	2R:3714246-3717355	2R:3717356-3720465	2R:2434119-2437303	0.0000	0.0000	(3,3)	3110	0	NA
Post5	3R:23784504-23787149	3R:23787150-23789795	3R:23490886-23493549	0.0000	0.0000	(2,2)	2646	0	NA
Post6	X:13069508-13075985	X:13075986-13082459	X:9960069-9966317	0.0000	0.0000	(1,1)	6476	0	NA
Post7	3L:6139113-6141328	3L:6141989-6144199	3L:5642666-5645059	0.0000	0.0000	(1,2)	2213.5	660	NA
Post8	3R:5510437-5517756	3R:5517757-5525642	3R:15817049-15824774(-)	0.0003	0.0000	(4,3)	7603	0	NA
Post9	2L:20442296-20451413	2L:20451414-20460527	2L:20012993-20022064	0.0010	0.0000	(3,3)	9116	0	NA
Post10	2R:7007474-7011226	2R:7011240-7014993	2R:5548919-5553813	0.0023	0.0000	(1,1)	3753.5	13	NA
Post11	2L:22071173-22072962	2L:22102566-22104351(-)	2L:21644922-21646603	0.0054	0.0021	(1,1)	1788	29603	region1
Post12	2L:11992238-11996148	2L:11996149-12000059	2L:11800219-11804142	0.0010	0.0022	(4,4)	3911	0	NA
Post13	X:8980939-8982165	X:8982166-8983401	X:7167799-7168382	0.0123	0.0044	(1,1)	1231.5	0	NA
Post14	X:7791319-7792508	X:7792509-7793694	X:6218797-6219758	0.0000	0.0071	(1,1)	1188	0	NA
Post15	3L:16588973-16594192	3L:16596653-16601883	3L:15933622-15938735	0.0092	0.0109	(2,2)	5225.5	2460	NA
Post16	2R:9293378-9298104	2R:9298105-9302842	2R:7751869-7756482	0.0028	0.0156	(2,2)	4732.5	0	NA
Post17	3R:15596923-15599055	3R:15601016-15603110	3R:5885559-5887804(-)	0.0045	0.0186	(1,1)	2114	1960	region2
Post18	X:19706416-19707385	X:19709760-19710733	X:15194358-15195350	0.0811	0.0250	(1,1)	972	2374	NA
Post19	X:13229824-13230415	2R:6709313-6709889(-)	X:10121156-10121741	0.0101	0.0309	(1,1)	584.5	-	region1
Post20	2L:3785212-3785664	2L:3785953-3786386	2L:3741960-3742405	0.0103	0.0358	(1,1)	443.5	288	NA
Post21	X:15234293-15236213	X:15236773-15238715	X:11757252-11759284	0.0329	0.0394	(1,1)	1932	559	region1
Post22	2L:14878773-14879923	2L:14881860-14882992	2L:14628312-14629433	0.0425	0.0508	(1,1)	1142	1936	region1
Post23	X:2319336-2319794	X:6846313-6846756(-)	super_0:17213325-17214005^a^	0.0109	0.0644	(1,1)	451.5	4526518	NA
Post24	3L:11124987-11125422	3L:11128095-11128536(-)	3L:10524349-10524814(-)	0.0427	0.0985	(1,1)	439	2672	region1
Post25	3R:18317472-18318043	3R:18318798-18319400	3R:18126604-18127193	0.1271	0.1539	(1,1)	587.5	754	NA

*L*: Average size of duplicated blocks in *D. melanogaster*.

*I*: The length of the region between duplicated blocks in *D. melanogaster*.

(*sX*, *sY*): The numbers of annotated coding genes in regions Xm and Ym, respectively.

The genomic locations of duplicated blocks are according to the *Drosophila melanogaster* genome 5.3 (dm3).

a Location is based on the *D. sechellia* genome.

**Table 2:** Summary of the Pre-Speciation Duplicated Blocks.

Block ID	*D. melanogaster*		*D. simulans* (*D. sechellia*)						
	Region X (Xm)	Region Y (Ym)	Ortholog X (Xs)	Ortholog Y (Ys)	*Ks*	*Ka*	(*s_X_*, *s_Y_*)	*L*	*I*
Pre1	2L:16849347-16850416	2L:16853903-16854977	2L:16534794-16535200	2L:16541726-16542150^b^	0.0000	0.0000	(1,1)	1072.5	3486
Pre2	X:3683952-3690380	X:3693058-3699472	super_4:3012729-3019165(-)^a^	super_1119:389-5078^a^ ^b^	0.0000	0.0023	(3,3)	6422	2677
Pre3	3L:22685251-22686898	3L:22689061-22690708(-)	super_11:2601985-2603895(-)^a^	super_11:2606124-2608055(-)^a^ ^b^	0.0000	0.0037	(2,2)	1648	2162
Pre4	3L:18581910-18583262	3L:18585405-18586760(-)	3L:17893431-17894750	3L:17896768-17899641(-)^b^	0.0042	0.0091	(1,1)	1354.5	2142
Pre5	3R:25747878-25748774	3R:25749499-25750395(-)	3R:25960042-25960744	3R:25956680-25957416(-)	0.0000	0.0116	(1,1)	897	724
Pre6	3L:14936820-14937480	3L:14938595-14939256	3L:14254850-14255498^b^	3L:14256922-14257574	0.0115	0.0129	(1,1)	661.5	1114
Pre7	X:3134181-3134665	X:3136385-3136856(-)	X:2222178-2222626	X:2225000-2225479(-)	0.0041	0.0138	(1,1)	478.5	1719
Pre8	X:18675644-18677917	X:18680352-18682599	X:14415283-14417484^b^	X:14419955-14422261	0.0059	0.0162	(1,1)	2261	2434
Pre9	2R:4597200-4597825	2R:4600913-4601538	2R:3245390-3246014	2R:3282879-3283504	0.0000	0.0170	(1,1)	626	3087
Pre10	2L:21199196-21199991	2L:21201493-21202309	2L:20782016-20782791	2h_random:1723429-1724213(-)	0.0178	0.0176	(1,1)	806.5	1501
Pre11	3L:19464698-19466005	3L:19468262-19469571(-)	3L:18791156-18792153(-)	3L:18798716-18799908(-)	0.0037	0.0186	(2,2)	1309	2256
Pre12	3R:12813834-12817362	3R:12818042-12821583	3R:8655806-8659366(-)	3R:8650764-8654343(-)	0.0007	0.0188	(2,2)	3535.5	679
Pre13	3L:15827810-15827986	3L:15829856-15830032	3L:15171528-15171704	3L:15173649-15173824	0.0000	0.0216	(1,1)	177	1869
Pre14	X:5836299-5838672	X:5839368-5841692	X:4561116-4564458	X:4565827-4567708	0.0534	0.0238	(1,1)	2349.5	695
Pre15	X:8350979-8351783	X:8359731-8360528	X:6642588-6643359	X:6650839-6651577	0.0123	0.0274	(1,1)	801.5	7947
Pre16	2R:13006207-13007808	2R:13012272-13013877(-)	2R:11744363-11745179	2R:11749685-11751291(-)	0.0029	0.0350	(1,1)	1604	4463
Pre17	3L:17826375-17826912	3L:17827921-17828452	3L:17166328-17166770	3L:17167222-17167763	0.0000	0.0454	(1,1)	535	1008
Pre18	3L:20314180-20315497	3L:20336039-20337356	3L:19685646-19686484	3L:19693453-19694794	0.0424	0.0501	(2,1)	1318	20541
Pre19	3R:9222524-9222886	3R:9223532-9223914(-)	3R:12209819-12210201	3R:12211108-12211448(-)	0.0097	0.0511	(1,1)	373	645
Pre20	2R:15172049-15172954	2R:15175259-15176162(-)	2R:13882402-13883275	2R:13885576-13886456(-)	0.0139	0.0522	(1,1)	905	2304
Pre21	X:15562204-15563201	X:15570521-15571497(-)	X:12056883-12057841	X:12065131-12065867(-)	0.0175	0.0524	(1,1)	987.5	7319
Pre22	2R:8395375-8398226	2R:8399198-8402038	2R:6871842-6874569	2R:6875636-6876682	0.0357	0.0661	(1,1)	2846.5	971
Pre23	3R:26312821-26313679	3R:26314655-26315514(-)	3R:25956680-25957545	3R:25959886-25960744(-)	0.0034	0.0724	(1,1)	859.5	975
Pre24	X:7828040-7828582	X:7829206-7829751	super_50:114946-115486^a^	super_50:116137-116682^a^	0.0000	0.0917	(1,1)	544.5	623
Pre25	3L:16312125-16312481	3L:16313104-16313481(-)	3L:15652016-15652408	3L:15653034-15653411(-)	0.0038	0.0920	(1,1)	367.5	622
Pre26	3L:11582669-11583177	3L:11583942-11584453(-)	3L:10957956-10958462	3L:10959470-10959980(-)	0.0111	0.1054	(1,1)	510.5	764
Pre27	3L:10883315-10883772	3L:10885115-10885575	3L:10276733-10277190	3L:10278539-10278996	0.0110	0.1133	(1,1)	459.5	1342
Pre28	3L:20492801-20494435	3L:20497115-20498755	3L:19842774-19844711	3L:19846946-19848585^b^	0.0389	0.1253	(1,1)	1638	2679
Pre29	X:1713912-1714554	X:1715962-1716602	X:1207032-1207410	X:1208614-1209209	0.2128	0.1539	(1,1)	642	1407
Pre30	2R:10634754-10635625	2R:10636619-10637480	2R:9399749-9400611	2R:9401599-9402459	0.0168	0.1680	(1,1)	867	993

See the legend of Table 1.

a Location is based on the *D. sechellia* genome.

b The block includes a pseudogene, which is functional in *D. melanogaster*.

We identified the orthologous regions of these duplicated blocks in the *D. simulans* and *D. sechellia* genomes, and the results are shown in Tables 1 and 2. For the 25 blocks in Table 1, there is only one orthologous region, while the orthologous regions of both the duplicated blocks are found in the *D. simulans* and/or *D. sechellia* genomes for the remaining 30 blocks (Table 2). The orthologs of Xm and Ym are denoted by Xs and Ys, respectively, in Table 2. The locations for Xs and Ys are those in the *D. simulans* genome if Xs and Ys are found in this species, otherwise the locations are those in the *D. sechellia* genome. The relative chromosomal locations and orientations of the blocks in the two species are consistent with each other for most of the duplicated blocks. Considering that it is very unlikely that the identical size of duplication occurred at the same genomic location and in the same orientation independently on the lineages leading to *D. melanogaster* and *D. simulans* (*D. sechellia*), it may be reasonable to consider that the duplicates in Table 2 were created by a single duplication event before the speciation of the two species. Therefore, these duplicates are referred to as “pre-speciation duplicates”. Note that the difference in orientation for Pre12 can be explained by a large inversion difference on chromosome 3R [28]. Pre5 and Pre11 are only exceptions, for which the possibilities of independent duplications cannot be ruled out although single duplication plus inversion will also explain them. In the following analysis, we treat all duplicated blocks in Table 2 as pre-speciation duplicates, but exclusion of Pre5 and Pre11 has very little effect on our conclusions. The duplicates in Table 1 are called “post-speciation duplicates” because they likely arose after the speciation of *D. melanogaster* and *D. simulans*.

For all post- and pre-speciation blocks, NJ trees on the basis of nucleotide divergence are constructed with *D. yakuba* homologs as an outgroup (Figure S1 and S2). The phylogenetic relationship is relatively simple for the post-speciation blocks (Figure S1): *D. melanogaster* has two copies while *D. simulans* and *D. sechellia* have one copy each, suggesting that the duplication events occurred after the speciation of *D. melanogaster* and the other two species. For the pre-speciation duplicated blocks, in most cases, phylogeny includes two duplicates in *D. melanogaster* and their orthologs in *D. simulans* and *D. sechellia* (Figure S2).

**Figure 2:**
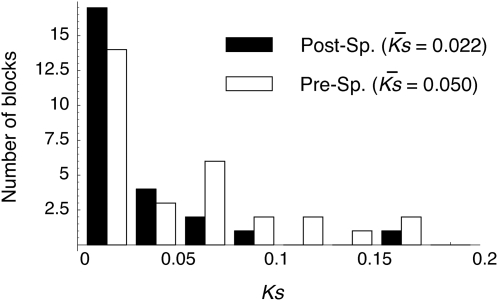
The distribution of synonymous divergence between duplicated blocks in *D. melanogaster*. *K̅* *_S_* is the average synonymous divergence for blocks with multiple coding genes. Post-Sp. and Pre-Sp. mean duplicates that arose after and before the speciation event of *D. melanogaster* and *D. simulans*, respectively.

Figure 2 shows the distributions of *K_S_* for the two classes of duplicated blocks. The overall distribution is L-shaped as reported by Lynch and Conery [13], mainly due to the excess of duplicated blocks with low *K_S_*. Almost all post-speciation blocks have *K_S_*<0.1 except for Post25. The tree for Post25 in Figure S1 shows that the duplicates in *D. melanogaster* are most closely related each other. It seems that the divergence is high only in synonymous sites in the coding region.

*K_S_* for the pre-speciation blocks are also low. If the two duplicated blocks have accumulated substitutions independently (*i.e.*, a molecular clock holds for the paralogous divergence), the expectation of *K_S_* for the pre-speciation blocks is larger than *K_Sspecies_*, which is the orthologous divergence at synonymous sites. The genome-wide average of *K_Sspecies_* is 0.12 [21]. Although there should be variation in *K_Sspecies_* across genes, our observation is quite unlikely under a molecular clock model, indirectly suggesting that those duplicated genes are undergoing concerted evolution by gene conversion.

The role of gene conversion can be directly and clearly documented by examining the shape of the gene tree of the duplicated genes. If the duplicated blocks X and Y in the two species are currently undergoing concerted evolution, the two paralogous regions in each species are more closely related than the orthologous pairs, as illustrated in Figure 1C. Without gene conversion, the orthologous pairs should be more closely related (Figure 1B). It is also possible that only one paralogous pair is undergoing concerted evolution while the other is not (Figure 1D). Based on this idea, we investigated the shapes of the trees in Figure S2, which is summarized in Table 3. Out of the 28 blocks to which the analysis can be applied (excluding two blocks with no outgroup sequence available), 14 exhibited evidence for gene conversion for both species (*i.e.*, the tree shape in Figure 1C), and evidence for gene conversion is obtained for either of the two species (*i.e.*, Figure 1D) for 10 blocks. It seems that the effect of gene conversion in *D. simulans* and *D. sechellia* is not as extensive as that in *D. melanogaster*, because nine of the 10 blocks have the Xm-Ym cluster (Table 3). However, this can be simply explained by the ascertainment bias of our sampling of duplicates: our sample is biased toward those with low paralogous divergence in *D. melanogaster*. No evidence for gene conversion is obtained in four blocks.

**Table 3:** Testing for Gene Conversion in the Post-Speciation Duplicated Blocks.

Block ID	Tree shape	Evidence for conversion*		*L*′	*j*	*k*	*k̅*	*P*
		*D.mel*	*D.sim*					
Pre1	NA	NA	NA	860	0	22	4.16	<0.0001
Pre2	(((**Xm**,**Ym**),Xs),Ys,yak)	yes	no	4631	0	209	5.41	<0.0001
Pre3	(((Xs,Ys),**Ym**),**Xm**,yak)	no	yes	1608	0	223	19.44	<0.0001
Pre4	((**Xm**,**Ym**),(Xs,Ys),yak)	yes	yes	927	0	27	1.21	<0.0001
Pre5	((**Xm**,**Ym**),(Xs,Ys),yak)	yes	yes	703	0	30	2.19	<0.0001
Pre6	(((**Xm**,**Ym**),Ys),Xs,yak)	yes	no	645	4	13	1.21	<0.0001
Pre7	((**Xm**,**Ym**),(Xs,Ys),yak)	yes	yes	434	1	52	5.25	<0.0001
Pre8	((**Xm**,**Ym**),(Xs,Ys),yak)	yes	yes	2137	2	108	7.71	<0.0001
Pre9	((**Xm**,**Ym**),(Xs,Ys),yak)	yes	yes	625	3	6	0.10	<0.0001
Pre10	((**Xm**,**Ym**),(Xs,Ys),yak)	yes	yes	757	4	26	2.04	<0.0001
Pre11	(((**Xm**,**Ym**),Xs),Ys,yak)	yes	no	992	0	52	2.20	<0.0001
Pre12	((**Xm**,**Ym**),(Xs,Ys),yak)	yes	yes	3460	0	145	6.87	<0.0001
Pre13	(((**Ym**,Ys),**Xm**),Xs,yak)	no	no	176	2	2	0.07	0.0026
Pre14	((**Xm**,**Ym**),(Xs,Ys),yak)	yes	yes	990	0	57	11.23	<0.0001
Pre15	((**Xm**,**Ym**),(Xs,Ys),yak)	yes	yes	709	0	60	8.61	<0.0001
Pre16	(((**Xm**,**Ym**),Xs),Ys,yak)	yes	no	817	5	9	0.46	<0.0001
Pre17	(((**Xm**,**Ym**),Ys),Xs,yak)	yes	no	427	15	8	2.99	0.0116
Pre18	(((**Xm**,**Ym**),Ys),Xs,yak)	yes	no	816	1	34	8.48	<0.0001
Pre19	(((**Xm**,**Ym**),Xs),Ys,yak)	yes	no	322	0	1	0.20	0.1824
Pre20	(((**Xm**,**Ym**),Ys),Xs,yak)	yes	no	872	7	26	4.27	<0.0001
Pre21	(((**Ym**,Ys),**Xm**),Xs,yak)	no	no	737	8	6	0.24	<0.0001
Pre22	((**Xm**,**Ym**),(Xs,Ys),yak)	yes	yes	969	1	38	3.19	<0.0001
Pre23	(((**Xm**,Ys),**Ym**),Xs,yak)	no	no	859	2	8	2.06	0.0013
Pre24	((**Xm**,**Ym**),(Xs,Ys),yak)	yes	yes	541	4	11	0.73	<0.0001
Pre25	((**Xm**,**Ym**),(Xs,Ys),yak)	yes	yes	351	1	5	0.32	<0.0001
Pre26	(((**Xm**,**Ym**),Ys),Xs, yak)	yes	no	499	4	27	3.42	<0.0001
Pre27	((**Xm**,**Ym**),(Xs,Ys),yak)	yes	yes	458	1	12	1.50	<0.0001
Pre28	((**Xm**,**Ym**),(Ys,Xs),yak)	yes	yes	1574	38	42	5.09	<0.0001
Pre29	NA	NA	NA	354	7	8	3.74	0.0373
Pre30	(((**Ym**,Ys),**Xm**),Xs,yak)	no	no	861	39	19	4.58	<0.0001

*: The presence of evidence for gene conversion in the *D. melanogaster* and *D. simulans* (*D. sechellia*) based on the tree shape analysis. The regions for which an outgroup sequence is available are analyzed. “yes” and “no” represent the presence and absence of evidence.

*L*′: the number of nucleotides used in the statistical analysis of the four sequence-alignments.

*j* and *k*: the numbers of type-N and type-C sites, respectively. *k*′ is the expectation of *k* (see text, especially Methods for details).

The power to detect evidence for gene conversion should increase if we perform a window-analysis of the tree shape. This is because the tree shapes in Figure S2 (also summarized in Table 3) reflect the *average* evolutionary relationship over the entire region (block). Therefore, this approach could potentially miss signatures of gene conversion when occurring only in local regions. In other words, the approach can detect evidence for gene conversion when it frequently occurs in most of the analyzed region. The results of the window analysis are also shown in Figure S2, where regions with red bar have tree shapes illustrated in Figure 1C (evidence for gene conversion in both species), while regions with blue bar have tree shapes illustrated in Figure 1B (no evidence for gene conversion). We observe that the tree shape changes across duplicated regions, indicating that different regions have different evolutionary histories. This is expected because gene conversion tracts should be much smaller than the duplicated regions. It is also suggested that there could be substantial local variation in the activity of gene conversion. Overall, there is evidence for extensive gene conversion. We found that in most of the analyzed blocks, the regions of red bar (*i.e.*, supporting the tree shape of Figure 1C) distribute along the entire region. All blocks investigated have at least one local region (window) supporting the tree shape of Figure 1C.

A drawback of this analysis is that the relative effect of other noises, including multiple mutations, would be large because phylogeny is constructed for short regions (windows). In other words, a small number of sites with multiple mutations could mimic the real evolutionary history of the duplicated blocks. Therefore, we apply a statistical test that incorporates the effect of multiple mutations. The null hypothesis is set such that the evolutionary history in the entire duplicated region follows the tree shape of Figure 1C, so that the observation could be explained without gene conversion when the effect of multiple mutations is taken into account. The *P*-value is the rejection probability of this null hypothesis; therefore, a smaller *P*-value indicates a stronger evidence for gene conversion.

**Figure 3:**
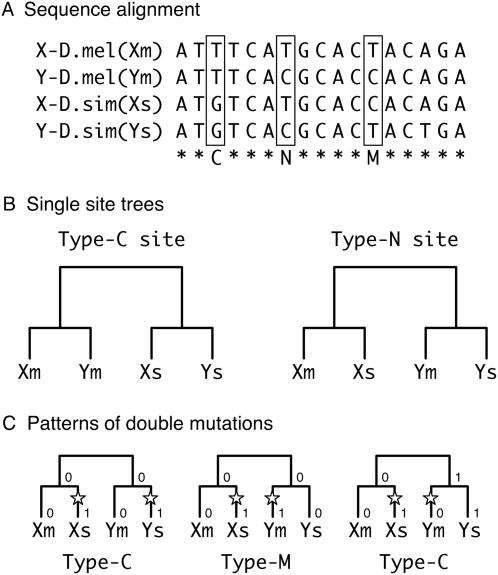
Illustrations to describe the analysis of informative sites in the alignment of the four sequences. (A) Example of the alignment of the four sequences. The types of informative sites are shown below the alignment: “C” and “N” are as defined in the text, and “M” represents a site that requires multiple mutations for explanation. (B) Relationships of the four sequences at type-C and type-N sites. (C) Patterns of double mutations. A double-mutated site is defined as one with a single substitution that has occurred since the speciation event in each of X and Y.

The statistical analysis is based on the alignment of the four sequences, Xm, Ym, Xs, and Ys (Figure 3). We focus on two types of informative sites in the alignment, denoted by type-C and type-N sites (Figure 3A). The former is a biallelic site at which the same nucleotide is shared by the two paralogous sequences in each species, while the latter is that at which the same nucleotide is shared by the two orthologous sequences (Figure 3A). A type-C site parsimoniously supports a tree with gene conversion (*i.e.*, the left tree in Figure 3B), while a type-N site supports a tree with no gene conversion (*i.e.*, the right tree in Figure 3B). Let *j* and *k* be the observed numbers of type-N and type-C sites, respectively. The presence of type-C sites (*k*>1) parsimoniously suggests that (at least a part of) the duplicated block experienced gene conversion, but multiple mutations could also explain it, especially when *k*≪*j*. The statistical test examines if the observed number (*k*) can be explained by multiple mutations assuming no gene conversion (see Methods). As shown in Table 3, the *P*-value is less than 0.05 for almost all pre-speciation blocks (29/30), most of which exhibit very strong evidence with *P*<0.0001. The exception is Pre19 for which only one informative site is available; thus, almost no statistical power is expected.

### Rate of Gene Duplication

We use our list of 1→2 duplications to estimate the rate of gene duplication. Note that our interest is in the long-term duplication rate, that is, the rate at which a duplicate arises by mutation and becomes fixed in the population. As mentioned in the Introduction, our focus is limited to two-copy duplicates to perform the fine-scale analysis at the DNA level. Therefore, the rate we estimate can be considered to be the rate at which a single-copy gene becomes two-copy duplicated genes. In this sense, the rate we are interested in is quantitatively different from those estimated in other articles [13],[22].

We have identified 63 gene duplications by which single-copy genes became two-copy genes. It was found that 31 of them are in the 25 post-speciation blocks, indicating that these 1→2 duplications occurred after the speciation of *D. melanogaster* and *D. simulans*, which was roughly 2.3 million years ago [29]. It can be estimated that a 1→2 duplication occurs every 0.074 million years, or the duplication rate per gene is 1.0×10^−9^, given that there are about 13,000 single-copy genes in the genome.

The advantage of this phylogeny-based method is that it is robust to the effect of gene conversion, which could cause a serious overestimation of gene duplication rate when estimated by counting duplicated genes with low divergence [15]. To investigate this effect of gene conversion, we estimated the 1→2 duplication rate following the method of Lynch and Conery [13]. We found 25 two-copy duplicated genes with synonymous divergence *K_S_*<0.01. Their ages should be smaller than 2.3×10^6^×0.01/0.12 = 1.9×10^5^ years. Thus, the divergence-based method produced the duplication rate per gene as 10.0×10^−9^, which was roughly 10 times larger than our estimate.

### Decay of Duplication Blocks

**Figure 4:**
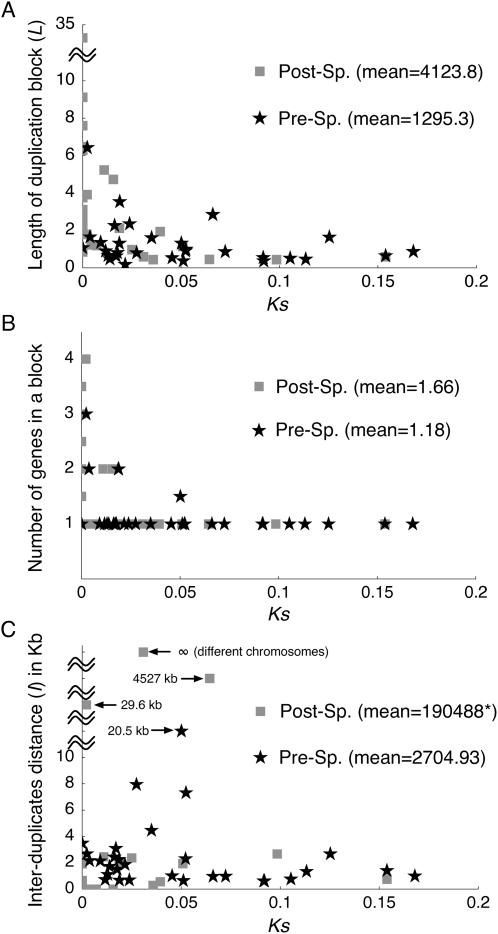
Decay of duplicated blocks. (A) Length of duplicated blocks (*L*) vs. synonymous divergence (*K_S_*). (B) Number of annotated genes vs. *K_S_*. (C) Length of intervening region (*I*) vs. *K_S_*.

Figure 4 displays the evolutionary changes in the size of duplicated blocks, the number of genes in each block, and the length of the intervening sequence between each pair. To understand their evolution over time, we used two methods to measure time. The first is the paralogous synonymous divergence (*K_S_*). Although *K_S_* is not a very good measure because of gene conversion (see above), theory predicts that *K_S_* at least shows a positive correlation with time since the duplication event [15]. Second, we directly compared the two classes of duplicates for the three characteristics of interest.

The relationship between *K_S_* and the block size is shown in Figure 4A. The sizes of duplicated blocks with *K_S_*<0.01 ranges from 1 kb to 35 kb, while the size is generally smaller than 2 kb for those with *K_S_*>0.1. *K_S_* and the block size show a strong negative correlation, and Pearson's correlation coefficient is *r* = −0.288, which is highly significant (*p*<0.0001, permutation test). It is also found that the average block size of the post-speciation blocks is significantly larger than that of the pre-speciation blocks (*p* = 0.0012, permutation test), indicating that young blocks are likely to be large.

Additionally, the number of genes (denoted by *s_X_* and *s_Y_* in Tables 1 and 2) in a block also decreases with increasing *K_S_* (*r* = −0.396, *p*<0.0001, permutation test). The average number of genes in the post-speciation blocks is significantly larger than that of the pre-speciation blocks (*p* = 0.0124, permutation test). It seems that young duplicated blocks have more genes. We found unannotated pseudogenes in several blocks, which resulted in *s_X_*≠*s_Y_* in Tables 1 and 2, suggesting that pseudogenization of redundant duplicated copies is underway. We also found that some orthlogs in *D. simulans* and/or *D. sechellia* have frameshift mutations (see Table 2).

Insertion/deletion is the mechanism to affect the size of duplicated blocks. Petrov et al. [30] reported that the deletion rate may be higher than the insertion rate in retrotransposons in the *D. melanogaster* genome. If this can be applied to duplicated regions, the biased pressure toward deletion would partly explain the observed decay of the sizes of duplicated blocks. The decay of the sizes of blocks could also be simply explained by technical limitations to identify the real duplicated regions. It may be easy to imagine that the accumulation of nucleotide mutations and small insertion/deletions around the edges of the duplicated regions could result in misidentification of the duplicated regions; usually, the “identifiable” region is smaller than the real region.

We propose that the decay of “identifiable” duplicated blocks can be enhanced by the combination of two opposing forces, mutation (including small indels) and gene conversion. Obviously, the former increases the divergence between duplicates, the latter decreases the divergence, and their balance determines the divergence between paralogs [31],[32],[33]. It may be reasonable to assume that the spatial distribution of the mutation rate would be roughly uniform, but there could be a substantial amount of local variation in the gene conversion rate. Because interlocus gene conversion is a kind of recombination event [2], we expect that the rate of paralogous synapses may be lower around the edges due to decreased sequence identity. As a consequence, the rate of gene conversion would be low around the edges. The divergence in these regions possibly increases more rapidly in comparison with that in regions far from the edges. This contrast in the pressure of homogenization by gene conversion could result in the misidentification of duplicated regions.

**Figure 5:**
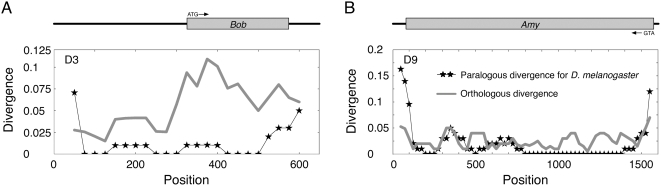
Distributions of the divergence between duplicated blocks, obtained by a window analysis with size 100 bp. (A) Pre6 block with the *Bob* genes. (B) Pre16 with the *Amy* genes.

This process predicts two outcomes. (i) The length of the intervening sequence between “identifiable” duplicated blocks (denoted by *I*) increases over time. This can be well documented if all duplication occur tandemly with no intervening region (*i.e.*, *I* = 0), but this is not the case in practice. Nevertheless, the prediction of increased intervening sequences may still be supported because all duplicated blocks with *I* = 0 are in the post-speciation class, and almost all (10/11) duplicated blocks with *I* = 0 have *K_S_*<0.01 (Tables 1 and 2). However, because many other mutational mechanisms are involved in the length evolution of intervening sequences, the relative contribution of the decay of duplicated block to the growth of intervening sequences may not be large. (ii) The second outcome would be seen in the distribution of the nucleotide divergence between duplicated blocks. The decay of the identifiable duplicated blocks could be visualized if the divergence is elevated around the edges when a high level of identity is observed in the middle of the block. Figure S2 illustrates the distribution of the paralogous divergence (blue line), which shows that many pre-speciation duplicated blocks have elevated divergence around the edges. Two examples with very clear patterns are picked up and shown in Figure 5. The first example is Pre6, which encompasses the *Bob* (Brother of Bearded) genes, and the second is Pre16 with the *Amy* (amylase) genes. In both, the divergence between paralogs is high around the edges of the identified blocks. Because the spatial distribution of orthologous divergence between the two species is not necessarily U-shaped in both the cases, the relaxation of negative selection outside the coding regions alone cannot explain the observation. The latter case is a typical example of duplicated genes with strong evidence for long-term concerted evolution by gene conversion [34],[33]. The two duplicates are shared by the *D. melanogaster* subgroup, indicating that the duplication occurred at least ∼10 million years ago. Such a long-term concerted evolution was achieved by frequent gene conversion: the rate has been estimated to be roughly 100 times higher than the synonymous mutation rate [32],[33],[35].

Thus, we have demonstrated that the size of “identifiable” duplicated blocks will shrink over time together, which can be explained by the accumulation of point mutations and ineffective gene conversion around the edges. The upshot is that it is difficult to know the real sizes of old duplicated blocks.

### Evolutionary Rate after Duplication

An acceleration in amino acid-changing substitutions (*K_A_*) after gene duplication is usually considered as a signature of neofunctionalization, although the relaxation of negative selection could also elevate the rate of non-synonymous substitutions. As shown in Tables 1 and 2, *K_A_* is smaller than *K_S_* in most cases, indicating the operation of purifying selection. Although several blocks have *K_A_*>*K_S_*, the ratio *K_A_*/*K_S_* is not significantly higher than 1.

Asymmetry of the evolutionary rate after gene duplication is another signature of neofunctionalization. Our data provide an opportunity to investigate the rate of molecular evolution in the original vs. derived copies. Since Ohno proposed his model of evolution of genetic novelty by gene duplication [1], this hypothesis has been challenged by many researchers [36],[37],[38]. Ohno's neofunctionalization model describes the process such that after a duplication, as long as one copy maintains the original function, the other is completely free from purifying selection. Therefore, Ohno's prediction has been tested for many species by looking at the symmetry (or asymmetry) of the evolutionary rate after gene duplication. However, those analyses did not specify which duplicates are original and which are derived copies. Here, with the availability of the genome sequences of *D. simulans* and *D. sechellia*, we were able to confidently define duplicates as original or derived copies for six of the post-speciation blocks (see Methods). We performed a relative rate test [39] by using the MEGA 3.1 program package [40] for genes in those six blocks, but we did not observe any significant trend in the acceleration of substitutions in the lineages of the original and derived copies.

### Signature of Selection for Neofunctionalization under the Pressure of Gene Conversion

**Figure 6:**
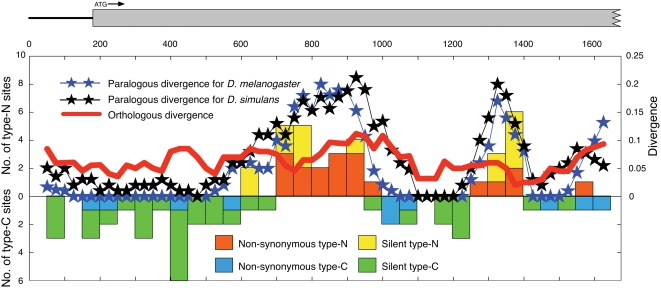
Distributions of divergences and type-C and type-N sites in Pre28, including the CG18281-CG17637 gene pairs in *D. melanogaster*. The orthologous divergence (*d*_0_) is the average of *d_Xm_*_−*Xs*_ and *d_Ym_*_−*Ys*_.

Teshima and Innan [24] recently proposed a new test for detecting signature of neofunctionalization. Using this simple non-parametric test, we performed a genome scan for recent neofunctionalization in *D. melanogaster*. The test can be best applied to relatively old duplicated blocks that are currently undergoing concerted evolution. In our data, the pre-speciation blocks with strong evidence for gene conversion should be suitable for this analysis. Because a simple search for locally diverged regions may capture false positives created in regions of less functional importance, we focused on the distributions of type-C and type-N sites. A cluster of type-N sites would be considered as a signature of neofunctionalization, which can be emphasized when there are many type-C sites in the surrounding regions of the cluster. A simple sliding-window analysis (see Methods) found such a pattern in one of the pre-speciation blocks. Figure 6 shows the distributions of type-C and type-N sites in Pre28 (below and above the horizontal axis, respectively). The observation is very well-consistent with the theoretical expectation with selection. There are two clusters of type-N sites, which are surrounded by regions with abundant type-C sites. A forward simulation (see Methods) showed that the probability that a peak of divergence with >15% appears in a 1600 bp region is very low (*P*<0.0001), suggesting that selection may be working at the two locations.

The two clusters are located in the coding regions of CG18281 (region X) and CG17637 (region Y), which belong to the major facilitator superfamily. The members in the major facilitator superfamily transport small solutes such as sugar and drugs in response to chemiosmotic ion gradients [41]. These two genes have conserved homologs among many metazoan organisms. A BLAST-based conserved domain search (CD search) showed that these two proteins contain arabinose or drug efflux domains of bacteria in their N-terminal regions [42].

Figure 6 also shows the distributions of the paralogous divergences for the two species. As expected, two peaks of divergence are observed at the same locations in both the distributions. The red line in Figure 6 is the distribution of *d_o_*, the divergence between the orthologous pairs, which is roughly flat across the region, indicating that the peaks of divergence are not due to the relaxation of purifying selection. This is also supported by an excess of non-synonymous type-N sites especially for the first peak around position 800 (14/20), indicating that the amino acid differences between duplicates may be preferred by selection. The distributions of the paralogous divergences for the two species are nearly identical, indicating that the peaks have been maintained by selection at least since the speciation of the two species.

This is also well-supported by phylogenetic trees in Figure S3. In the regions excluding the two peaks, the two paralogs in *D. melanogaster* are closely related to each other (Figure S3C). In contract, the tree for the first peak is consistent with the species tree (Figure S3A). The branch lengths in the tree in Figure S3A are overall longer than those in Figure S3B, suggesting an accelerated evolutionary rate in the region around the first peak. A similar pattern is also observed for the second peak, although the resolution of the tree is not very clear because the region is short (Figure S3B).

## Discussion

### Gene Duplication and Gene Conversion

The pattern of recent 1→2 gene duplications in the *D. melanogaster* genome was investigated with special attention to interlocus gene conversion. Our fine-scale analysis with careful visual inspections enabled accurate identification of duplicated blocks. The orthologous parts of those duplicated blocks were also identified in the *D. simulans* and *D. sechellia* genomes, by which we were able to clearly classify most blocks into post- and pre-speciation duplicated blocks. Our analysis demonstrated that a number of duplicated blocks undergo concerted evolution by gene conversion. Almost all pre-speciation duplicated blocks exhibited strong signatures of gene conversion (Table 3, Figure S2).

Gene conversion and unequal crossingover are usually considered the major mechanisms of concerted evolution. In this study, we focused only on gene conversion because unequal crossingover is not relevant. Our fine-scale identification of recent duplicated blocks showed that the synteny around the duplicated blocks are very well-conserved among *D. melanogaster*, *D. simulans* and *D. sechellia*, indicating that there is no evidence of unequal crossingover.

The decay of duplicated blocks over time was observed. We found that (1) the length of duplicated blocks is large for young duplicates (post-speciation blocks), (2) young duplicated blocks include more genes, (3) all duplicated blocks with no intervening sequences (*I* = 0) belong to the post-speciation class. In addition to biased deletion rate, which may be possible for *D. melanogaster* [30], we postulate that the degeneration of gene conversion around the edges enhances the divergence between duplicates, causing the misidentification of the real duplicated region; usually, the “identifiable” region is smaller than the real region. Our hypothesis is supported by the elevated paralogous divergence around the edges of duplicated regions as shown in Figures 5 and S2.

Thus, we provided several lines of evidence that gene conversion plays a crucial role after gene duplication in the *D. melanogaster* genome. Although most of the duplicated blocks analyzed in this study were located close together on the same chromosome, interlocus gene conversion can occur between different chromosomes. By looking at the polymorphism data in a pair of duplicated genes located on chromosomes 3 and X, Arguello et al. [43] showed clear evidence that the pair has been undergoing long-term concerted evolution by gene conversion. Polymorphism data analysis is much more powerful to detect interlocus gene conversion, and there are a number of duplicated gene pairs with strong signatures of recent gene conversion in *D. melanogaster* [33],[35]. It seems that interlocus gene conversion is a genome-wide phenomenon. Therefore, its effect should be taken in account in any kind of evolutionary analysis of gene duplication.

### Rate of Gene Duplication

We estimated the 1→2 gene duplication rate to be 1.0×10^−9^ per gene per year by using a phylogeny-based method. The method is robust to the effect of gene conversion, which is a great advantage. In contrast, a divergence-based method [13], which uses information from only a single genome, is very sensitive to gene conversion because it reduces the divergence between duplicated genes. We found that the divergence-based method provides an estimate of gene duplication rate about 10 times higher than that provided by the phylogeny-based method. The degree of overestimation is not as serious as in yeast, for which overestimation by the divergence-based method is about two orders of magnitude [11]. It should be note that the original estimate of Lynch and Conery is 2.3×10^−9^ per gene per year, which is only twice higher than ours even when they included small multigene families with sizes up to five. The reason for this is that they found only 10 duplicated gene pairs with *K_S_*<0.01 probably because of the incompleteness of the *D. melanogaster* genome at the time.

This study focuses only on 1→2 duplications because our primary purpose was to perform a fine-scale analysis at the DNA level including non-coding regions, which has not been done in previous large-scale analysis in *Drosophila* [21],[22]. In this sense, this study is different from others, including that of [13],[22], and [21], who analyzed gene families with various sizes. The rates of duplication (as defined above) depend on the size of the multigene family. The duplication rate of single-copy genes (*i.e.*, 1→2 duplication rate) should be lower than the rates of larger families (*e.g.*, 2→3, 3→4,… duplication rates), when selection is working on copy number. For example, if over-expression by a duplicated extra copy is deleterious, the extra copy is subject to negative selection, and this selective pressure is stronger for single-copy genes [44]. Although Hahn et al. [22] reported lineage-specic expansion of gene families, we did not observe such expansion in our data, indicating that the copy-number evolution in small families is more stable than that in large ones. Nevertheless, the estimate of Hahn et al. [22] is 1.0×10^−9^ per gene per year, which is quantitatively consistent with ours. This is because their estimate is based on net copy size changes over a long evolutionary time, so that it does not reflect some duplications canceled out by losses. Our estimate (1.0×10^−9^ per gene per year) is quantitatively more consistent with an estimated rate of new gene formation through DNA-level duplication by Yang et al. [45]. Their estimate (0.12×10^−9^) is several times lower than ours because they ignore tandem duplications. They found that most of those events are 1→2 duplications. It may be possible to extend our analysis to a larger gene family although technically more difficult [46], but description of such an analysis is beyond the scope of this article.

Note that we define the duplication rate as the rate at which a single-copy gene is duplicated and fixed in the population. Although our estimates assumed that all identified duplicated blocks are fixed in the *D. melanogaster* population, it is possible that some of them are still polymorphic (*i.e.*, copy-number polymorphism). If so, our estimates would be overestimated. If we exclude duplicates with too low *K_S_* (say, *K_S_*<0.01), our estimate turns out to be 0.4×10^−9^ per year, which can be considered as the lower limit of our estimate because this treatment might be too drastic: all duplicates with *K_S_*<0.01 are considered to be polymorphic. Indeed, only two of our post-duplicates are found to be polymorphic in a recent survey of copy-number variation by Emerson *et al.* [47], but this number may be underestimated because of their experimental strategy: Because Emerson *et al.* designed their research to map the regions of copy-number variation on the reference genome of *D. melanogaster*, it might not be optimized to detect copy-number variation in the reference genome itself.

Here, we arbitrarily defined duplicated genes as those with synonymous divergence less than 0.2. This definition is to cover duplicate pairs that could potentially exchange DNA sequences frequently by gene conversion. This cutoff value should not be unreasonable, according to our previous theoretical work [15], together with the observation in yeast [11]: *K_S_* for duplicated genes with evidence for gene conversion in yeast is usually less than 0.2. Our results are robust to this arbitrary cutoff value. For example, there is a very minor quantitative change in the estimate of gene duplication rate when the cutoff value is set as 0.3 because there are very few duplicated gene pairs with 0.2<*K_S_*<0.3.

### Selection after Gene Duplication

Neofuntionalization is one of the most important selective processes after gene duplication. To infer the action of natural selection, we first focused on the synonymous and nonsynonymous divergences (*K_S_* and *K_A_*) between duplicated genes, but we found no strong signature of selection for neofunctionalizations. There could be at least two reasons for this. First, such *K_S_*−*K_A_* analysis works best for relatively long-term molecular evolution, during which a substantial number of nucleotide substitutions accumulate. Therefore, the methods would not have sufficient statistical power for our data with recent duplicated genes, especially when active gene conversion between duplicated genes retards the paralogous divergence.

More importantly, gene conversion complicates the neofunctionalization process at the DNA level. When the duplicated genes undergo concerted evolution by gene conversion, which should be the case for many of the duplicated genes we analyzed, selection does not automatically result in the acceleration of nonsynonymous substitutions in the entire gene. The acceleration of substitutions will be limited to a narrow region around the target; therefore, *K_S_*−*K_A_*-based methods using the divergence in the entire gene should result in a lack of power. Instead, Teshima and Innan [24] suggested a possibility to focus on the spatial distribution of the divergence to detect signature of recent neofunctionalization. According to this idea, we found a strong signature in a pair of transporter genes (CG18281 and CG17637). This result indicates the promising possibility for applying this method as a genome scan for signatures of selection for neofunctionalization in other species. The advantage of our method is that it is possible to infer what parts of the genes are subject to selection.

## Methods

### Identification of Duplicated Blocks

*Drosophila* genome Release 3.1 (dm3) was used for the identification of duplicated genes. A total of 13,165 non-redundant protein sequences were in the database. All protein sequences were used as queries to search against all the others by using the BLASTP program with a cutoff value of *e*<10^−10^. We filtered out pairs of protein sequences with lower similarity than the criteria of Gu et al. [48], which is the protein identity *α*>0.3 if the alignable region *β*>150 bp, otherwise *α*≥0.06+4.8 *β* ^0.32[1+exp(*β*/1000)]^.

The duplicated genes detected in this screening process were aligned by using ClustalW [49]. The nucleotide divergence was estimated by using the method of Li-Pamilo-Bianchi [50],[51], and gene pairs with *K_S_*>0.2 were screened out. In this analysis, we use 63 duplicated genes. Genes with no homologs with *K_S_*<0.2 are considered as single-copy genes, and we found that the *D. melanogaster* genome has 12959 single-copy genes.

We identified the duplicated genomic regions (blocks) that involved those duplicated genes, using the BLASTN algorithm followed by visual inspection. The duplicated blocks were located on the latest version (Release 5.3; dm3) of the *D. melanogaster* genome, with the annotation data at the UCSC genome browser website (http:// genome.ucsc.edu/). For these duplicated blocks, their orthologs were searched in other species in the *D. melanogaster* subgroup (Figure 1). All aligned sequences are provided in Dataset S1.

### Phylogenetic Analysis

The DNA sequences of duplicated blocks in *D. melanogaster* and their orthologs were aligned together with an outgroup sequence from *D. yakuba* by using ClustalW [49]. Pairwise nucleotide distances [Kimura's distance, 52] were computed, from which an NJ tree was constructed (Figures S1 and 2).

### Inferring the Original and Derived States of the Duplicated Blocks

For the post-speciation duplicated blocks, it may be possible to infer which of the duplicates the original copy was, if the intervening sequence between the duplicates is relatively long (*i.e.*, *I*≫0). The intervening sequence was searched against the genome sequence of *D. simulans* (or *D. sechellia*). If the sequence has homology to the upstream of the *D. simulans* homolog, the downstream copy of *D. melanogaster* would be the original copy, and vice versa.

### Statistical Test for Detecting Local Gene Conversion

For each block, we first estimated the number of nucleotide substitutions per site between the two orthologous pairs, *p*_0_. Given this estimate, we consider *k̅*, the expected number of type-C sites in the duplicated block under a simple two-allele model with 0 and 1. The expected number of sites at which each of the X and Y regions experienced a mutation since speciation is roughly given by 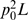, where *L* is the length of the duplicated block. At such a double-mutated site, the resultant pattern of the two alleles (0 and 1) depends on the branches on which the mutations occurred. The left and middle trees in Figure 3C consider cases where the two duplicated regions had the same allele, 0, at the speciation event. In the left tree, both the two mutations occurred in the lineages leading to the same species (*i.e.*, *D. simulans* in this example), so that the current allelic status for (X_m_, X_s_, Y_m_, Y_s_) is (0, 1, 0, 1) and the site becomes a type-C site. On the other hand, in the middle tree, the two mutations occurred in the lineages leading to different species (*i.e.*, in this example, in the *D. simulans* lineage at X and in the *D. melanogaster* lineage at Y), resulting in (X_m_, X_s_, Y_m_, Y_s_) = (0, 1, 1, 0). This pattern cannot be explained by a single mutation even with gene conversion and is referred to as a type-M site in Figure 3A. Thus, because the probabilities that a mutation occurs in the two lineages are half at both X and Y, a double-mutated site becomes a type-C site with probability 1/2 when X and Y had the same allele at the speciation event. Similar logic holds for the case where X and Y had different alleles at the speciation event, and the probability to become a type-C site is again 1/2 (see Figure 3C). Therefore, the expected number of type-C sites is given by 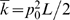. Our statistical test examines whether the observed number of type-C sites is significantly larger than this expectation, that is, the *P*-value is given by
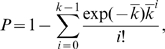
(1)assuming the Poisson distribution of mutations.

For simplicity, we employed a two-allele model, although the real sequence has four nucleotides. This method underestimates the *P*-values because the probability that a double-mutated site appears as a type-C site is much smaller than 1/2: in most cases, it becomes a triallelic site. Thus, our treatment is conservative in terms of detecting gene conversion.

### Detecting Signature of Selection

To detect signatures of selection, we used a sliding window approach. We set the window size = 200 bp. For each window, the numbers of type-C and type-N sites are computed, and compared with those in the surrounding regions (200 bp in each direction). In practice, a 2×2 contingency table is obtained and Fisher's exact *P*-value is computed. With a cutoff of *P*<0.0001, we found two peaks of the paralogous divregence, and both of them are in Pre28. A forward simulation was performed following Teshima and Innan [15], and it was found that a peak of divergence (>15% in a 200 bp window) appears in a 1600 bp region with probability <0.0001.

## Supporting Information

Figure S1 Phylogenetic analysis of post-speciation duplicated blocks. NJ trees of the orthologs in the *D. melanogaster* subgroup using the entire DNA sequences of duplicated blocks are shown.
(1.16 MB PDF)

Figure S2 (A) Phylogenetic analysis of pre-speciation duplicated blocks. NJ trees of the orthologs in the *D. melanogaster* subgroup using the entire DNA sequences of duplicated blocks are shown. (B) Window analysis of the spatial distribution of the tree structure and orthlogous and paralogous divergences. The window size is 100 bp. The regions with the tree shape in Figure 1B (no evidence for gene conversion) are represented by blue bars at the top of the panel, and those with the tree shape in Figure 1C (evidence for gene conversion in both the two species) are represented by red bars. Gray bars represent the regions with other tree shapes including the one in Figure 1D, and the regions with no outgroup data (i.e., *D. yakuba* and *D. erecta*) are shown in blank. The positions of type-N and type-C sites are presented by blue and red circles, respectively. The distribution of the divergence between the paralogs in *D. melanogaster* is shown by the blue curve, while that of the orthologous divergence between *D. melanogaster* and *D. simulans* is shown by the red curve. Window analysis was not applied to Pre1 and Pre29 because of the lack of data of the *D. yakuba* data.
(5.46 MB PDF)

Figure S3 The distributions of codon adaptation index (CAI, Sharp and Li 1987 Nucleic Acids Res. 15, 1281–1295) for single-copy genes (open circles) and for our duplicates (bar graph).
(0.06 MB PDF)

Dataset S1 Alignment data.
(0.20 MB ZIP)
