# Supplementary material for: Duplication and Gene Conversion in the Drosophila melanogaster Genome
Source: PLoS Genet. 2008 Dec 12;4(12):e1000305. doi: 10.1371/journal.pgen.1000305 (PMC2588116; doi:10.1371/journal.pgen.1000305)

Figure S1 — Post-speciation block 1

A) Duplication block in melanogaster subgroup (DNA sequence, Kimura's distance)

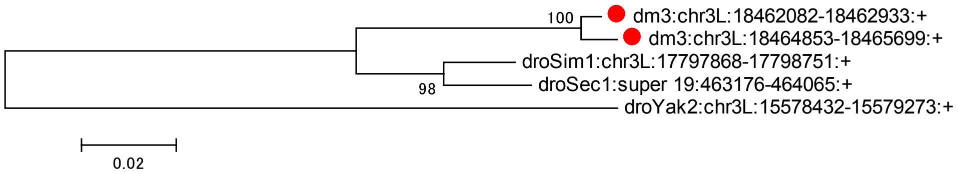

Figure S1 — Post-speciation block 2

A) Duplication block in melanogaster subgroup (DNA sequence, Kimura's distance)

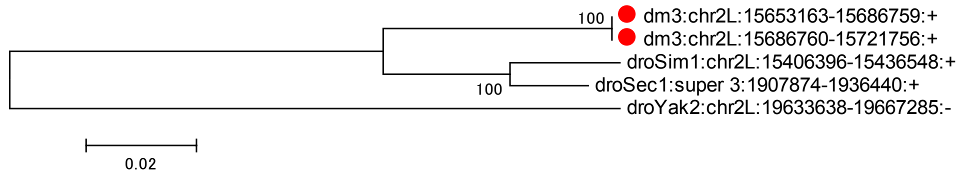

Figure S1 — Post-speciation block 3

A) Duplication block in melanogaster subgroup (DNA sequence, Kimura's distance)

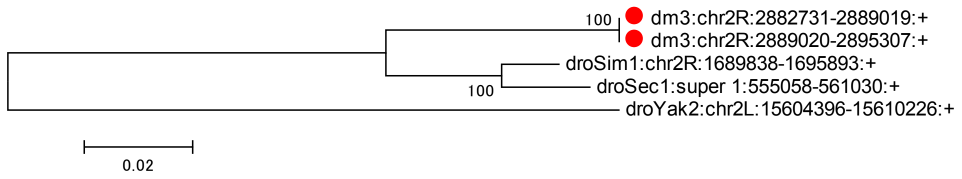

Figure S1 — Post-speciation block 4

A) Duplication block in melanogaster subgroup (DNA sequence, Kimura's distance)

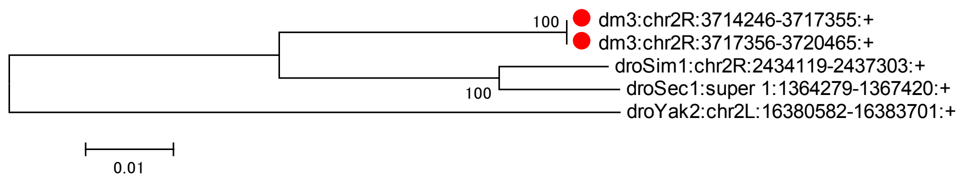

Figure S1 — Post-speciation block 5

A) Duplication block in melanogaster subgroup (DNA sequence, Kimura's distance)

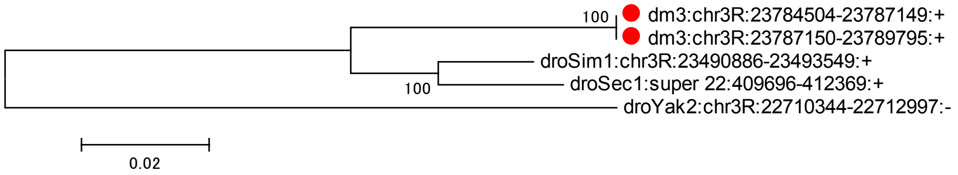

Figure S1 — Post-speciation block 6

A) Duplication block in melanogaster subgroup (DNA sequence, Kimura's distance)

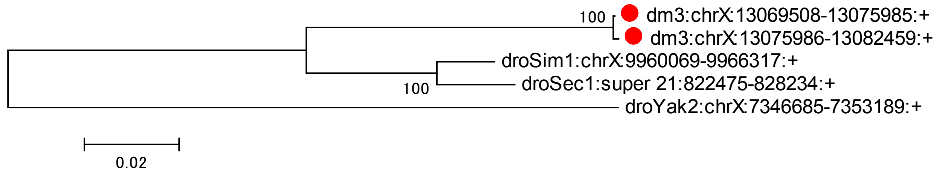

Figure S1 — Post-speciation block 7

A) Duplication block in melanogaster subgroup (DNA sequence, Kimura's distance)

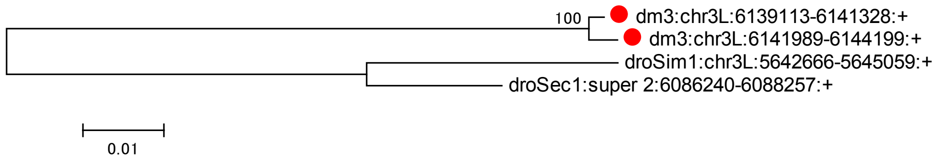

Figure S1 — Post-speciation block 8

A) Duplication block in melanogaster subgroup (DNA sequence, Kimura's distance)

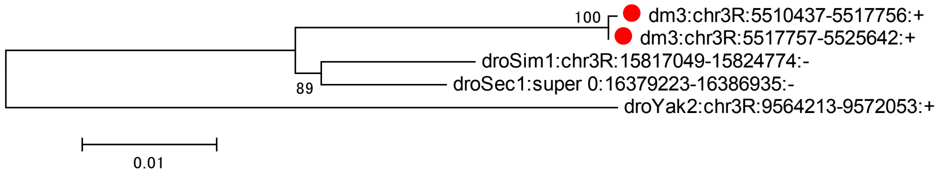

Figure S1 — Post-speciation block 9

A) Duplication block in melanogaster subgroup (DNA sequence, Kimura's distance)

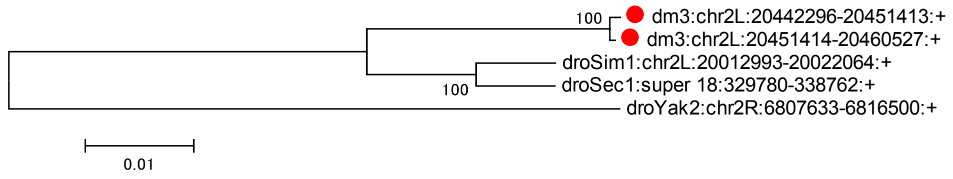

Figure S1 — Post-speciation block 10

A) Duplication block in melanogaster subgroup (DNA sequence, Kimura's distance)

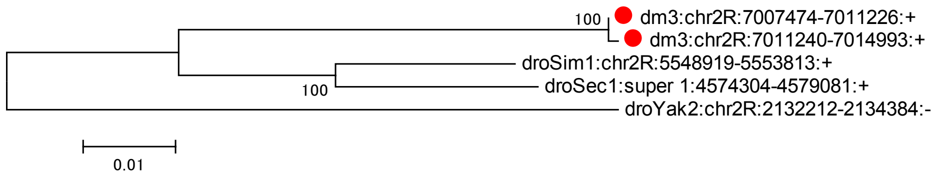

Figure S1 — Post-speciation block 11

A) Duplication block in melanogaster subgroup (DNA sequence, Kimura's distance)

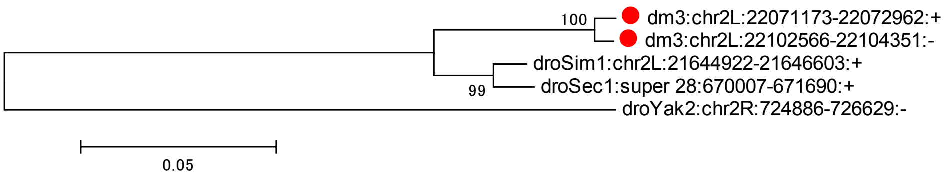

Figure S1 — Post-speciation block 12

A) Duplication block in melanogaster subgroup (DNA sequence, Kimura's distance)

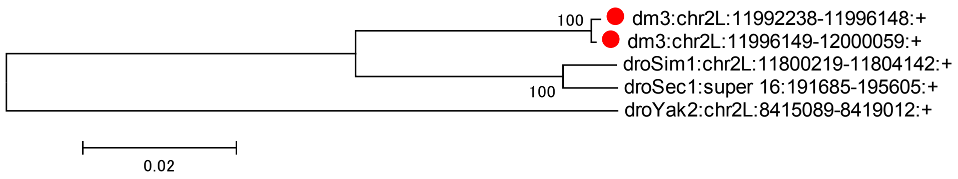

Figure S1 — Post-speciation block 13

A) Duplication block in melanogaster subgroup (DNA sequence, Kimura's distance)

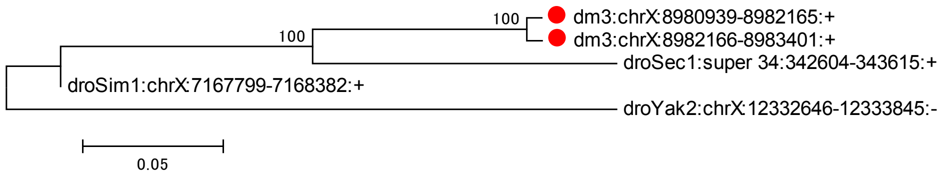

Figure S1 — Post-speciation block 14

A) Duplication block in melanogaster subgroup (DNA sequence, Kimura's distance)

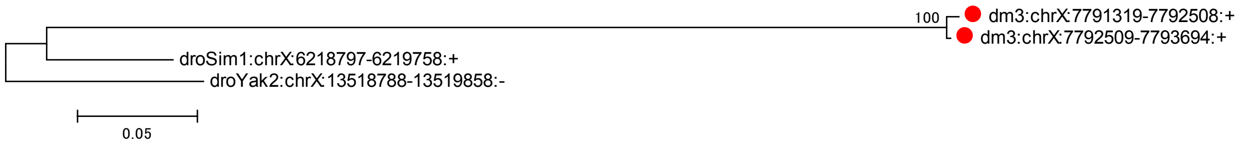

Figure S1 — Post-speciation block 15

A) Duplication block in melanogaster subgroup (DNA sequence, Kimura's distance)

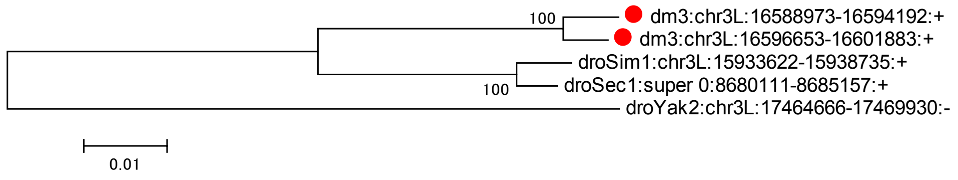

Figure S1 — Post-speciation block 16

A) Duplication block in melanogaster subgroup (DNA sequence, Kimura's distance)

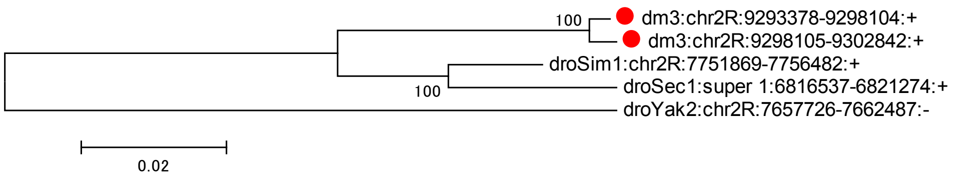

Figure S1 — Post-speciation block 17

A) Duplication block in melanogaster subgroup (DNA sequence, Kimura's distance)

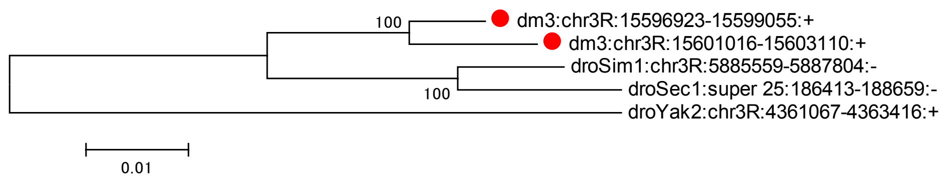

Figure S1 — Post-speciation block 18

A) Duplication block in melanogaster subgroup (DNA sequence, Kimura's distance)

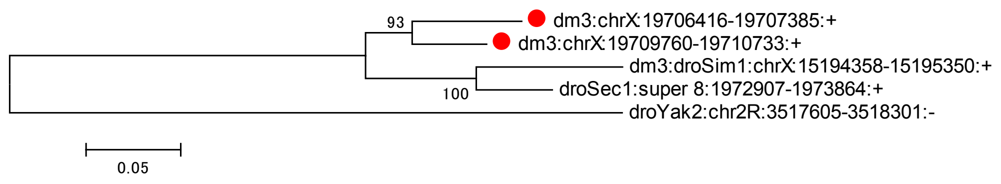

Figure S1 — Post-speciation block 19

A) Duplication block in melanogaster subgroup (DNA sequence, Kimura's distance)

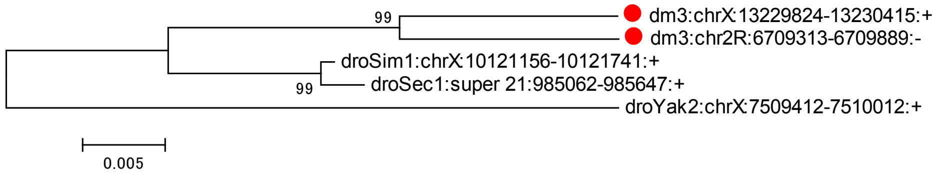

Figure S1 — Post-speciation block 20

A) Duplication block in melanogaster subgroup (DNA sequence, Kimura's distance)

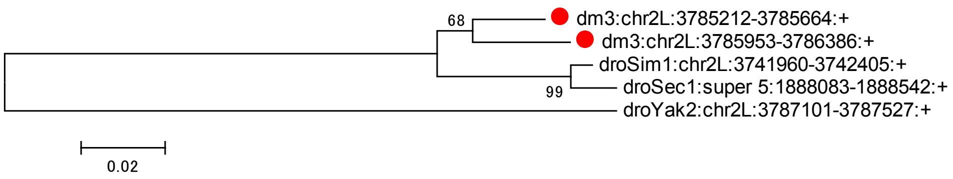

Figure S1 — Post-speciation block 21

A) Duplication block in melanogaster subgroup (DNA sequence, Kimura's distance)

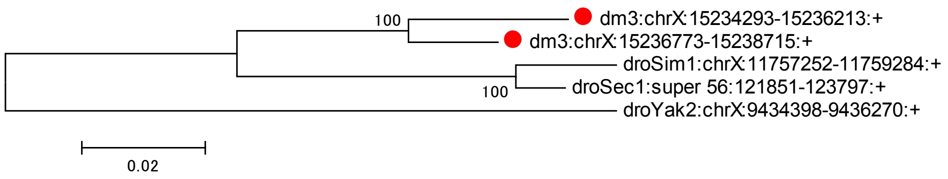

Figure S1 — Post-speciation block 22

A) Duplication block in melanogaster subgroup (DNA sequence, Kimura's distance)

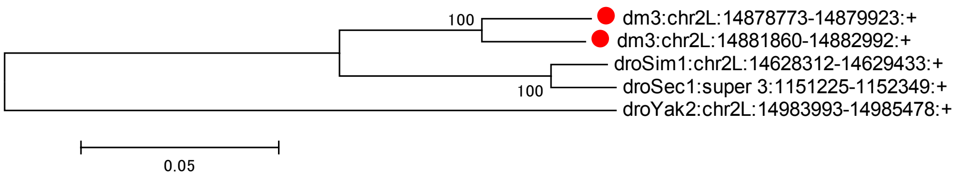

Figure S1 — Post-speciation block 23

A) Duplication block in melanogaster subgroup (DNA sequence, Kimura's distance)

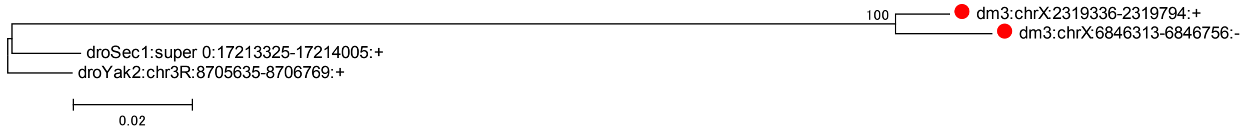

Figure S1 — Post-speciation block 24

A) Duplication block in melanogaster subgroup (DNA sequence, Kimura's distance)

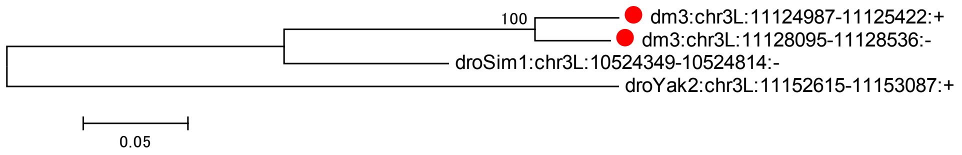

Figure S1 — Post-speciation block 25

A) Duplication block in melanogaster subgroup (DNA sequence, Kimura's distance)

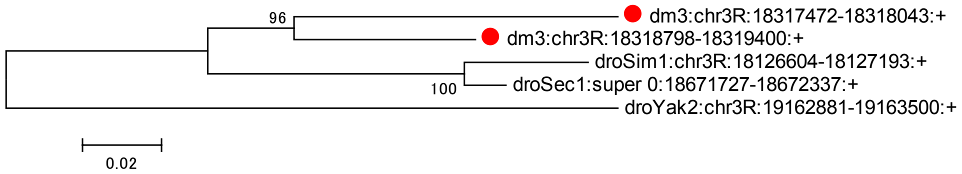

Supplement: Figure S1 — Phylogenetic analysis of post-speciation duplicated blocks. NJ trees of the orthologs in the D. melanogaster subgroup using the entire DNA sequences of duplicated blocks are shown. (1.16 MB PDF) [file pgen.1000305.s001.pdf]
