# Supplementary material for: Duplication and Gene Conversion in the Drosophila melanogaster Genome
Source: PLoS Genet. 2008 Dec 12;4(12):e1000305. doi: 10.1371/journal.pgen.1000305 (PMC2588116; doi:10.1371/journal.pgen.1000305)

Figure S2 — Pre-speciation block 1

A) Duplication block in melanogaster subgroup (DNA sequence, Kimura's distance)

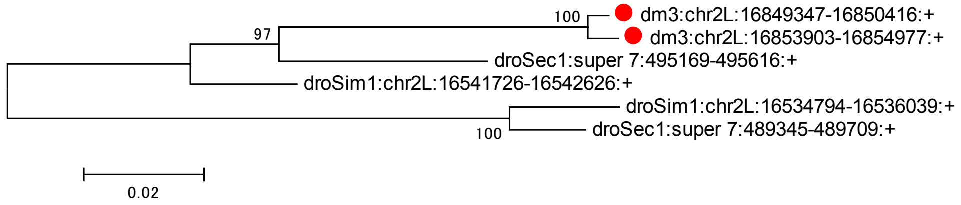

Figure S2 — Pre-speciation block 2

A) Duplication block in melanogaster subgroup (DNA sequence, Kimura's distance)

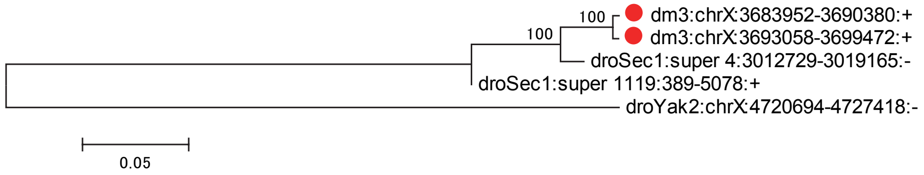

B) Window plot

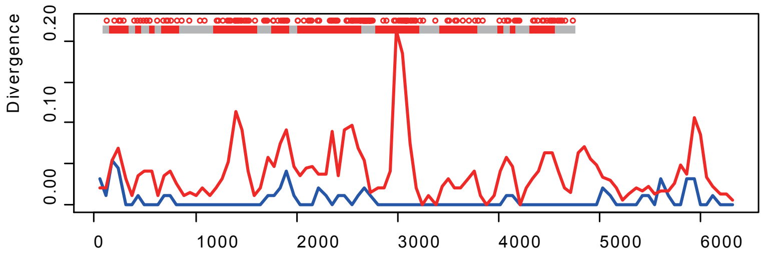

Figure S2 — Pre-speciation block 3

A) Duplication block in melanogaster subgroup (DNA sequence, Kimura's distance)

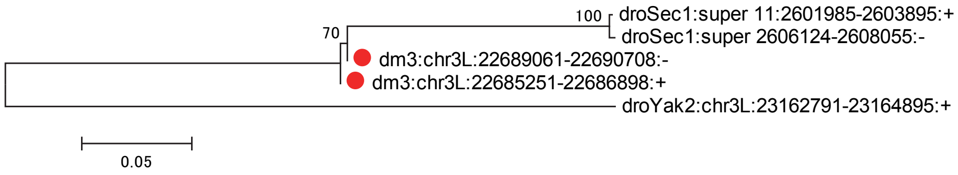

B) Window plot

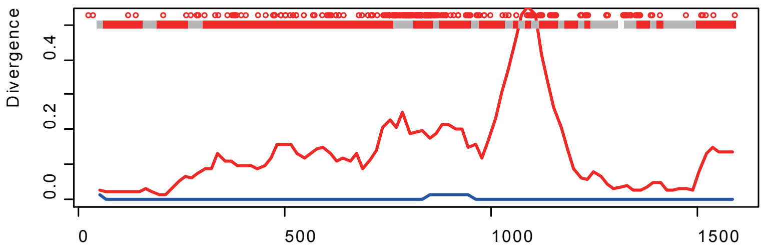

Figure S2 — Pre-speciation block 4

A) Duplication block in melanogaster subgroup (DNA sequence, Kimura's distance)

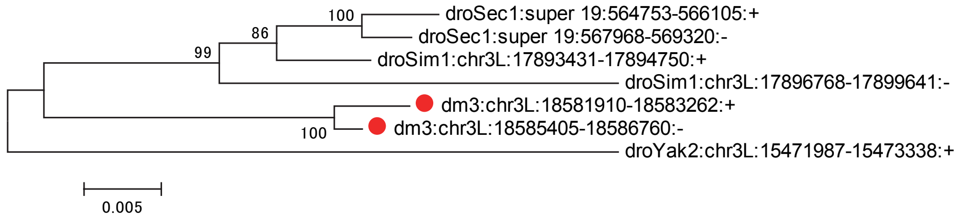

B) Window plot

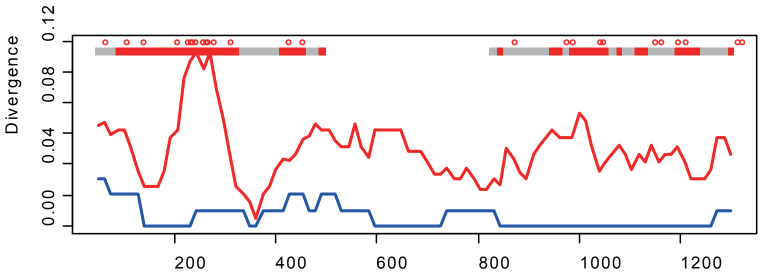

Figure S2 — Pre-speciation block 5

A) Duplication block in melanogaster subgroup (DNA sequence, Kimura's distance)

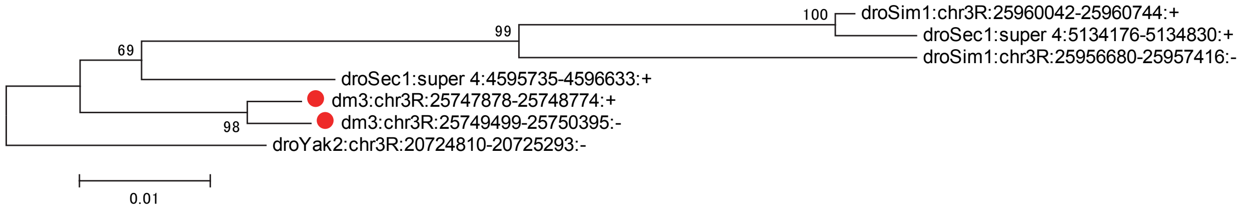

B) Window plot

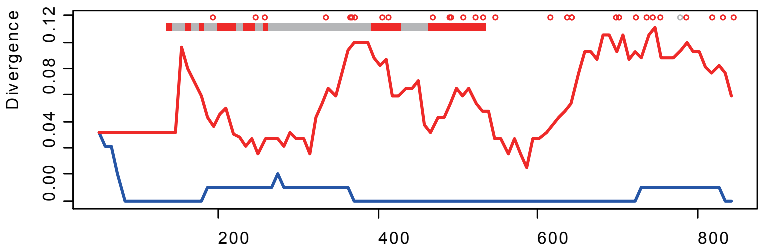

Figure S2 — Pre-speciation block 6

A) Duplication block in melanogaster subgroup (DNA sequence, Kimura's distance)

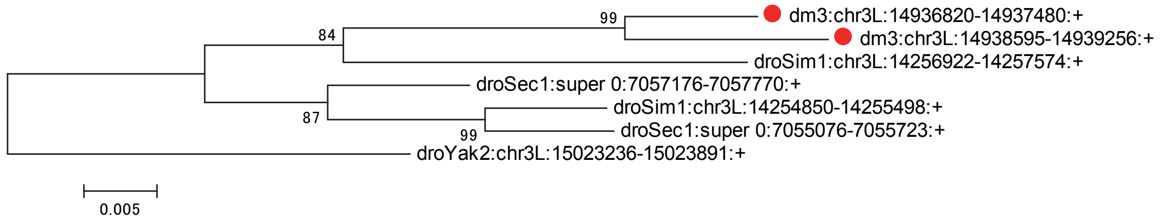

B) Window plot

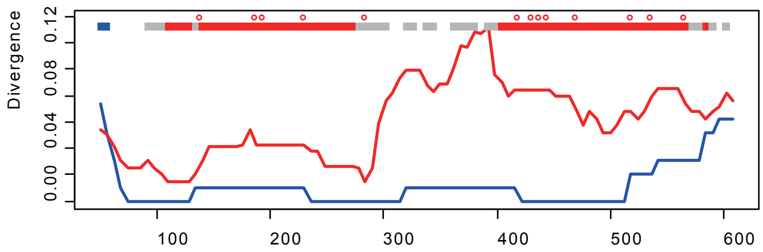

Figure S2 — Pre-speciation block 7

A) Duplication block in melanogaster subgroup (DNA sequence, Kimura's distance)

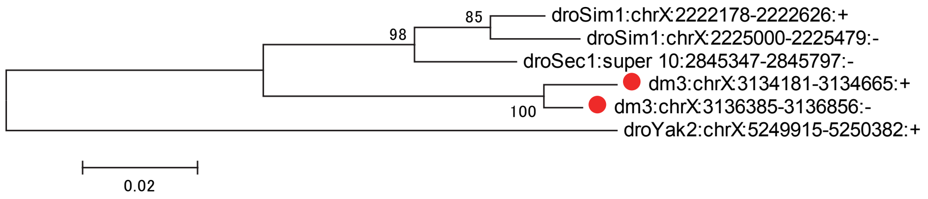

B) Window plot

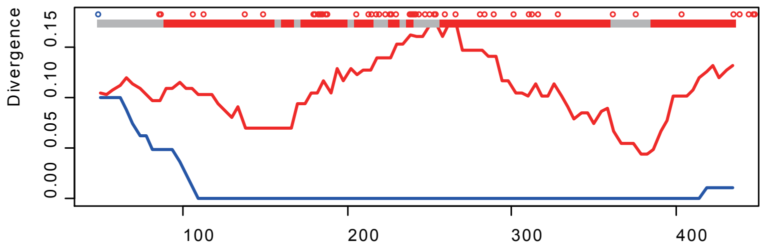

Figure S2 — Pre-speciation block 8

A) Duplication block in melanogaster subgroup (DNA sequence, Kimura's distance)

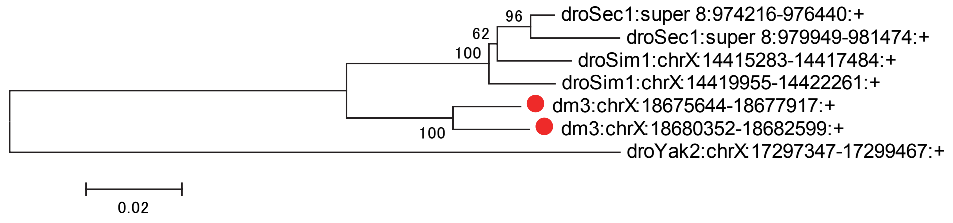

B) Window plot

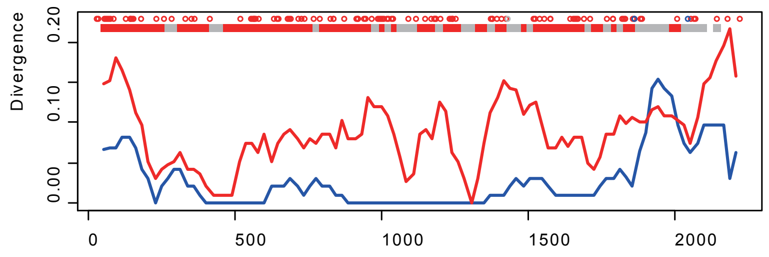

Figure S2 — Pre-speciation block 9

A) Duplication block in melanogaster subgroup (DNA sequence, Kimura's distance)

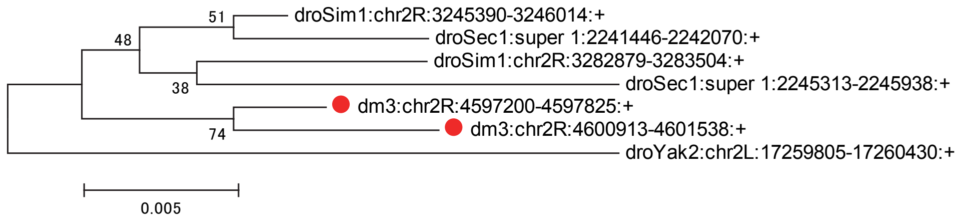

B) Window plot

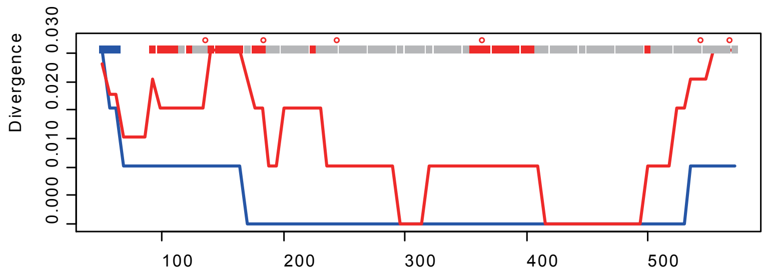

Figure S2 — Pre-speciation block 10

A) Duplication block in melanogaster subgroup (DNA sequence, Kimura's distance)

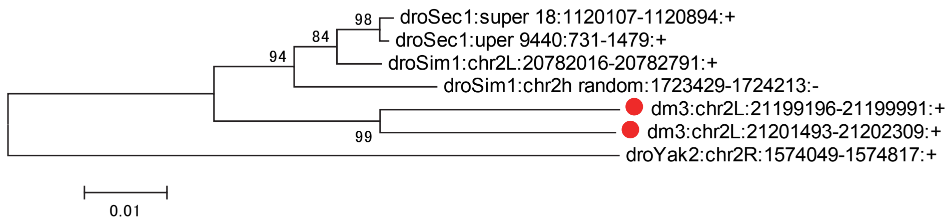

B) Window plot

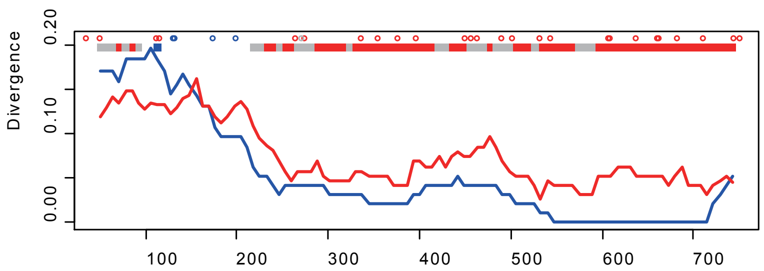

Figure S2 — Pre-speciation block 11

A) Duplication block in melanogaster subgroup (DNA sequence, Kimura's distance)

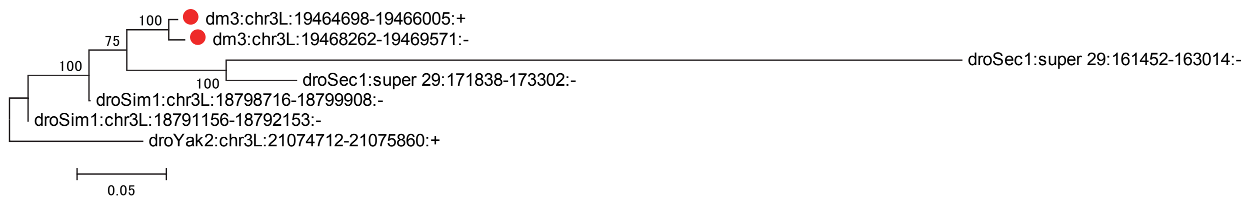

B) Window plot

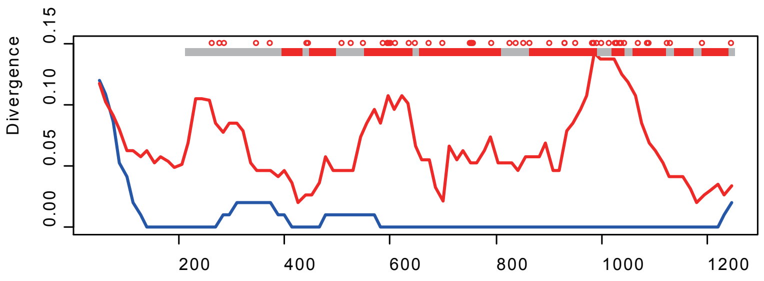

Figure S2 — Pre-speciation block 12

A) Duplication block in melanogaster subgroup (DNA sequence, Kimura's distance)

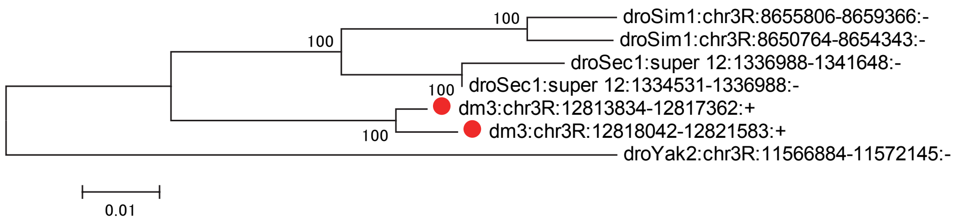

B) Window plot

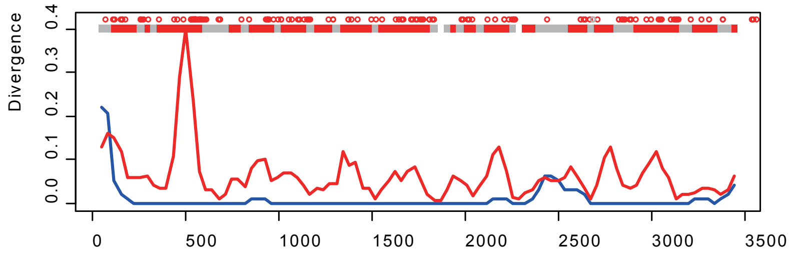

Figure S2 — Pre-speciation block 13

A) Duplication block in melanogaster subgroup (DNA sequence, Kimura's distance)

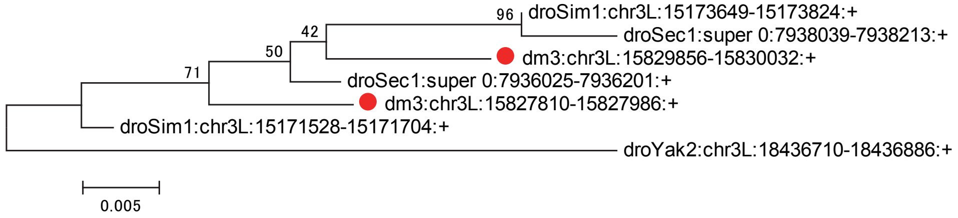

B) Window plot

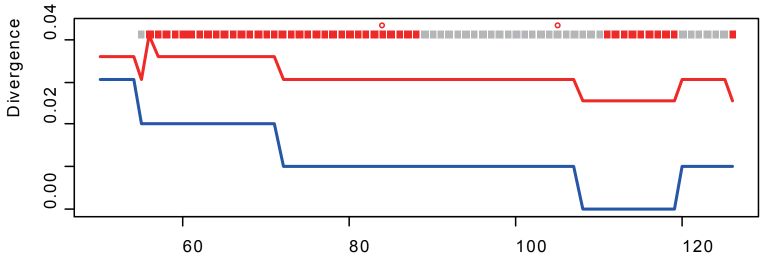

Figure S2 — Pre-speciation block 14

A) Duplication block in melanogaster subgroup (DNA sequence, Kimura's distance)

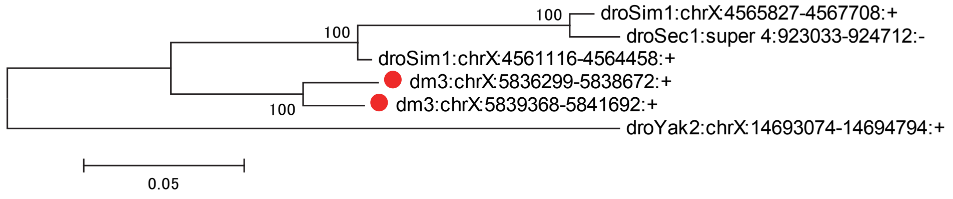

B) Window plot

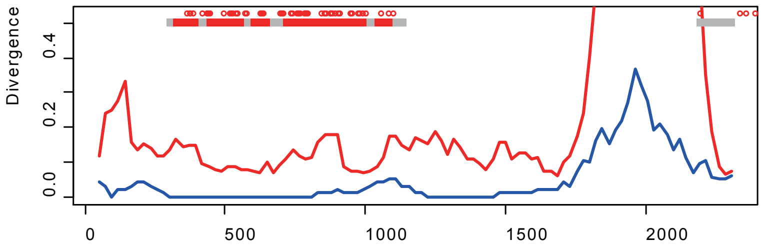

Figure S2 — Pre-speciation block 15

A) Duplication block in melanogaster subgroup (DNA sequence, Kimura's distance)

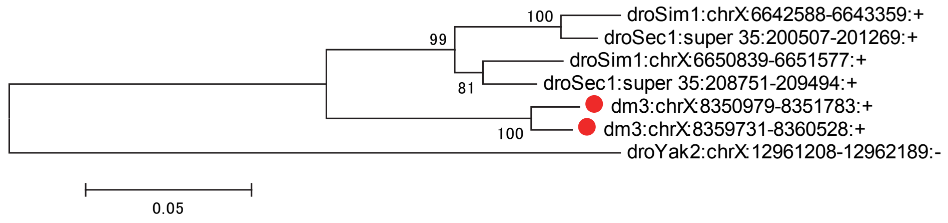

B) Window plot

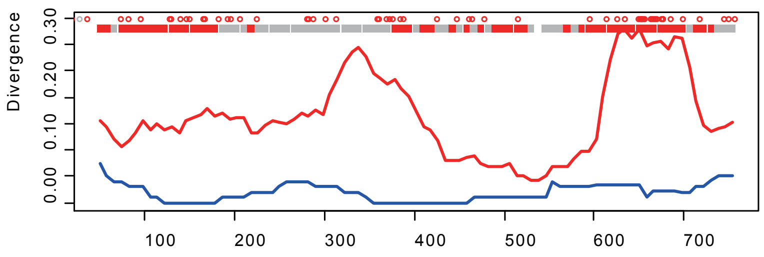

Figure S2 — Pre-speciation block 16

A) Duplication block in melanogaster subgroup (DNA sequence, Kimura's distance)

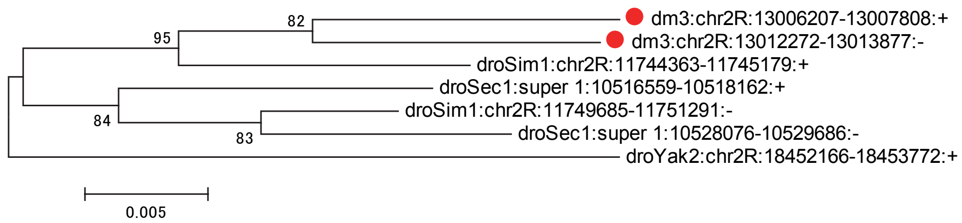

B) Window plot

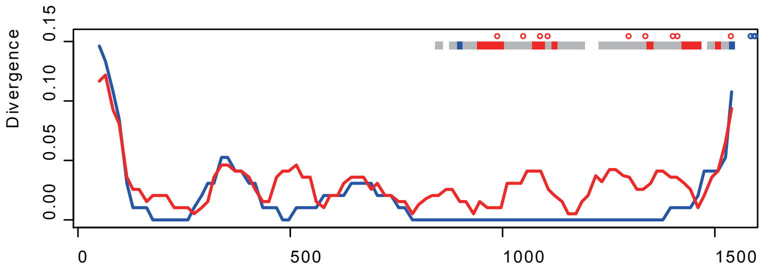

Figure S2 — Pre-speciation block 17

A) Duplication block in melanogaster subgroup (DNA sequence, Kimura's distance)

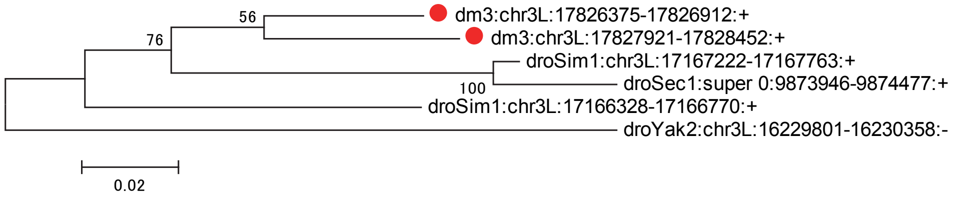

B) Window plot

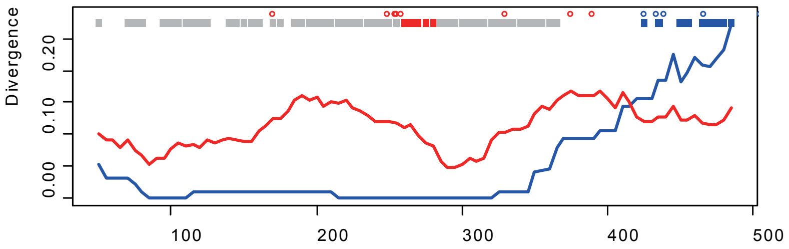

Figure S2 — Pre-speciation block 18

A) Duplication block in melanogaster subgroup (DNA sequence, Kimura's distance)

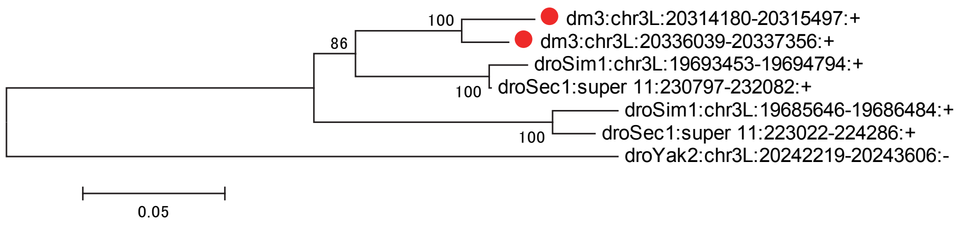

B) Window plot

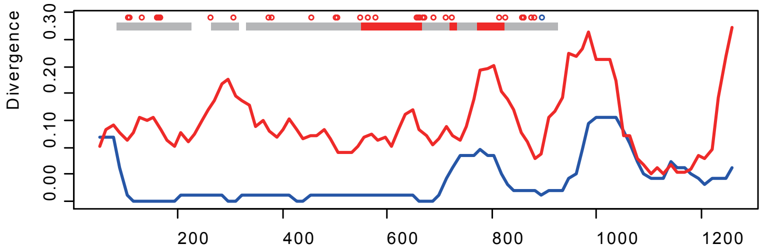

Figure S2 — Pre-speciation block 19

A) Duplication block in melanogaster subgroup (DNA sequence, Kimura's distance)

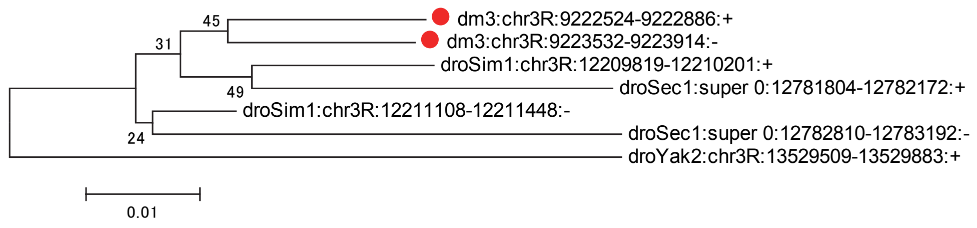

B) Window plot

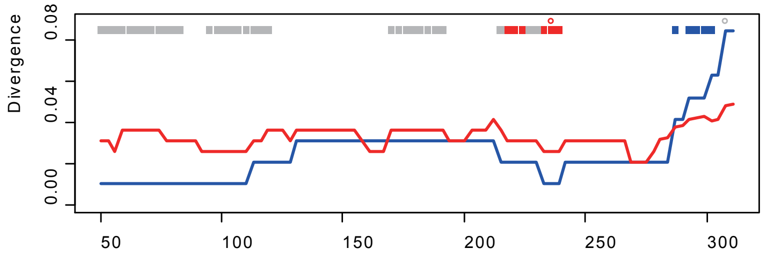

Figure S2 — Pre-speciation block 20

A) Duplication block in melanogaster subgroup (DNA sequence, Kimura's distance)

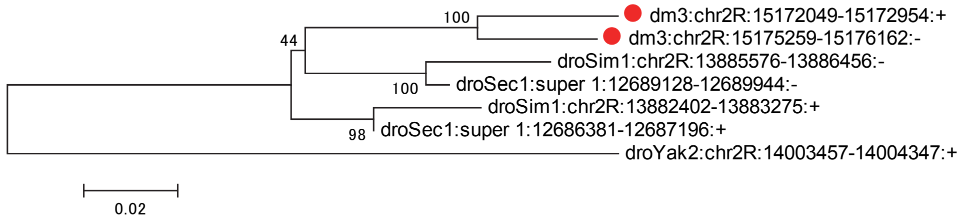

B) Window plot

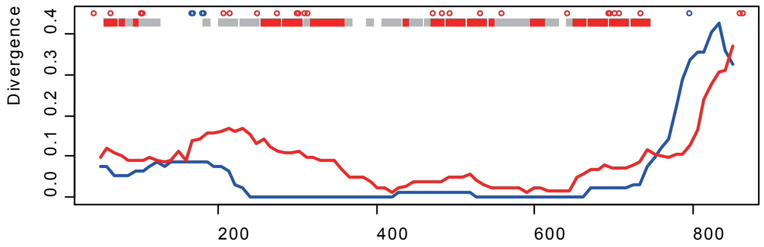

Figure S2 — Pre-speciation block 21

A) Duplication block in melanogaster subgroup (DNA sequence, Kimura's distance)

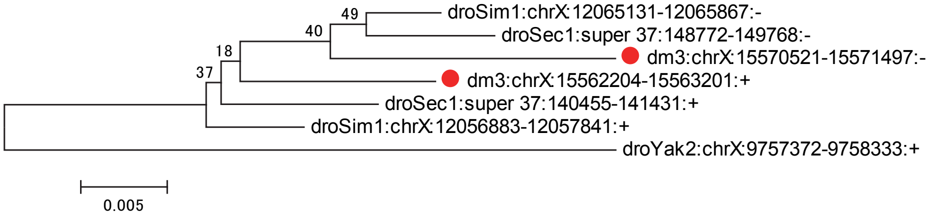

B) Window plot

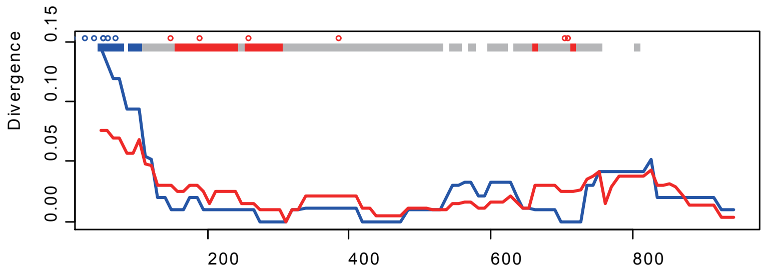

Figure S2 — Pre-speciation block 22

A) Duplication block in melanogaster subgroup (DNA sequence, Kimura's distance)

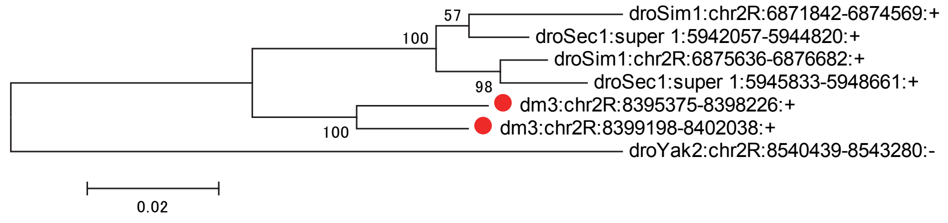

B) Window plot

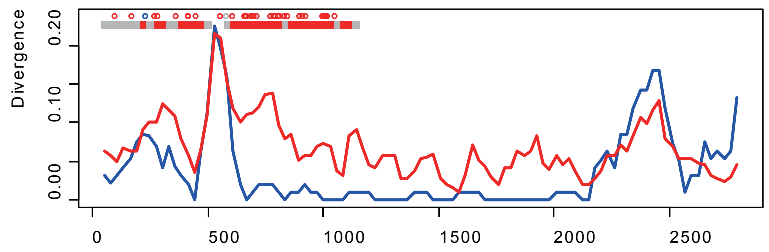

Figure S2 — Pre-speciation block 23

A) Duplication block in melanogaster subgroup (DNA sequence, Kimura's distance)

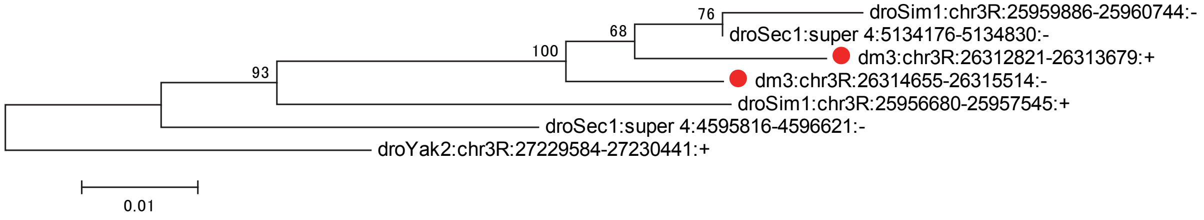

B) Window plot

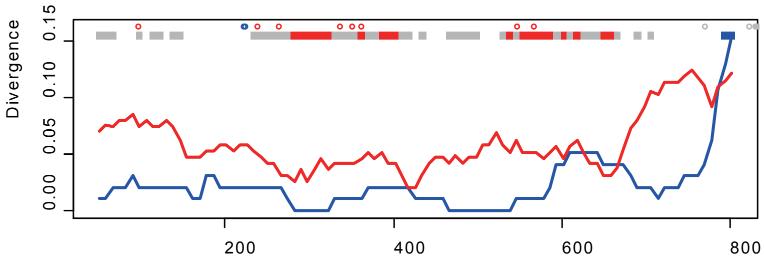

Figure S2 — Pre-speciation block 24

A) Duplication block in melanogaster subgroup (DNA sequence, Kimura's distance)

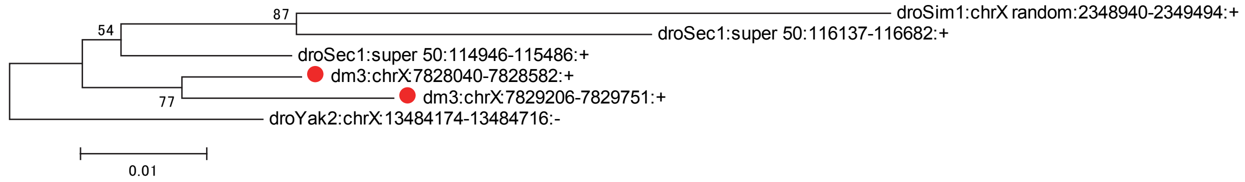

B) Window plot

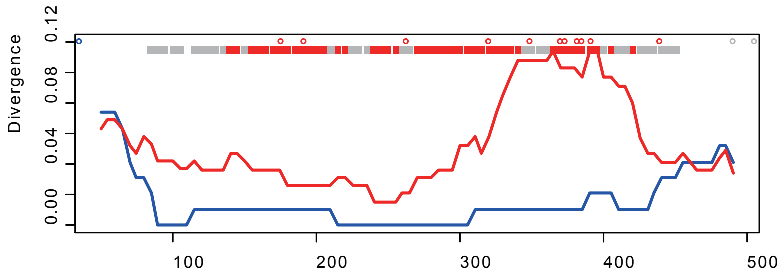

Figure S2 — Pre-speciation block 25

A) Duplication block in melanogaster subgroup (DNA sequence, Kimura's distance)

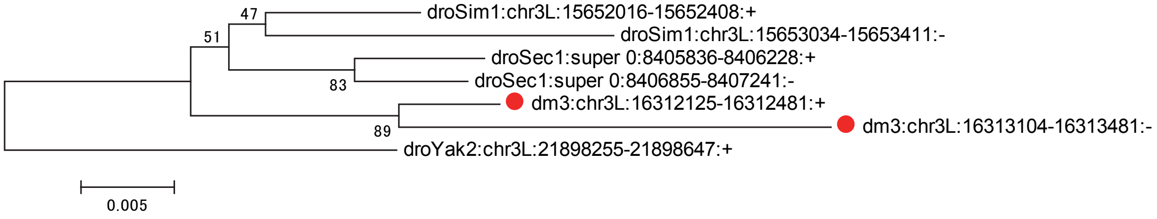

B) Window plot

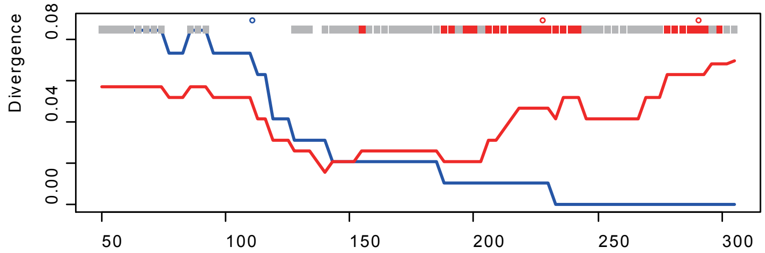

Figure S2 — Pre-speciation block 26

A) Duplication block in melanogaster subgroup (DNA sequence, Kimura's distance)

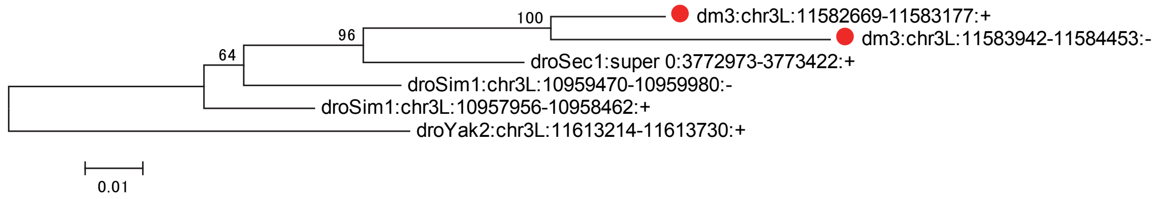

B) Window plot

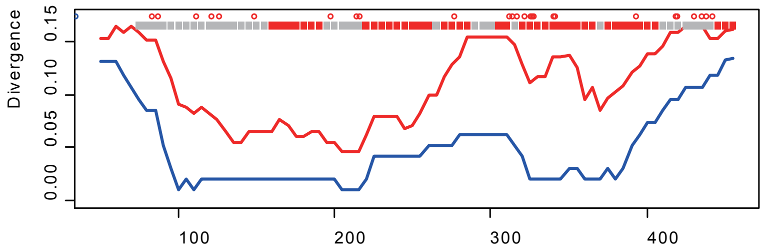

Figure S2 — Pre-speciation block 27

A) Duplication block in melanogaster subgroup (DNA sequence, Kimura's distance)

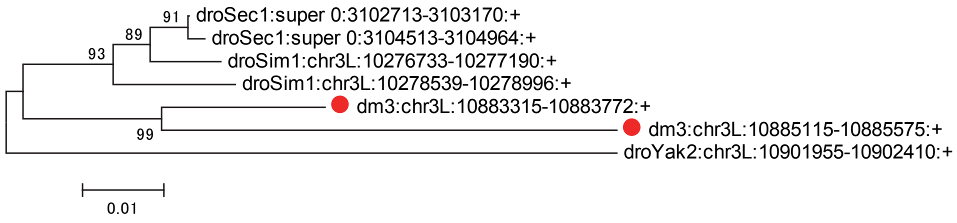

B) Window plot

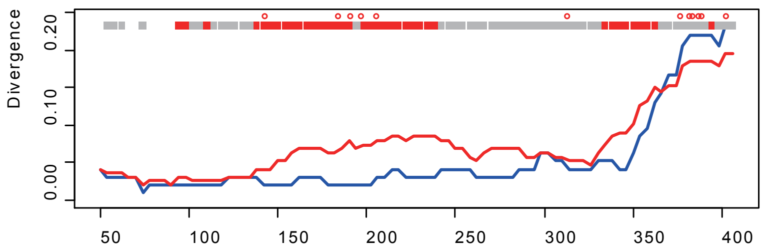

Figure S2 — Pre-speciation block 28

A) Duplication block in melanogaster subgroup (DNA sequence, Kimura's distance)

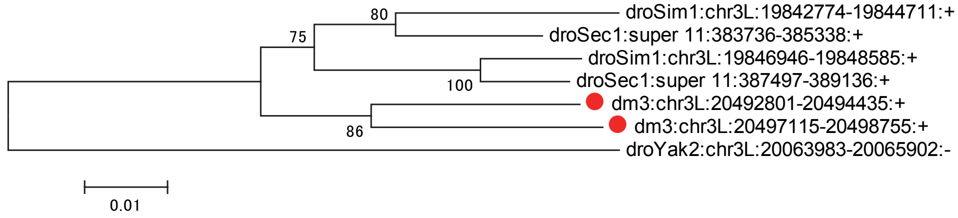

B) Window plot

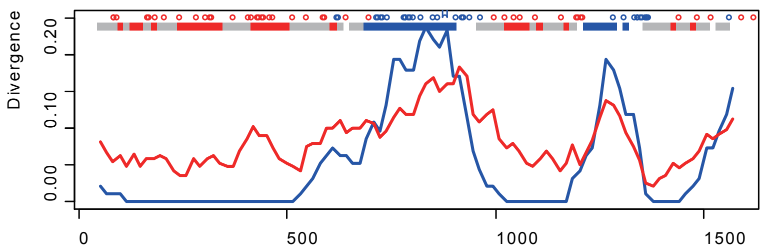

Figure S2 — Pre-speciation block 29

A) Duplication block in melanogaster subgroup (DNA sequence, Kimura's distance)

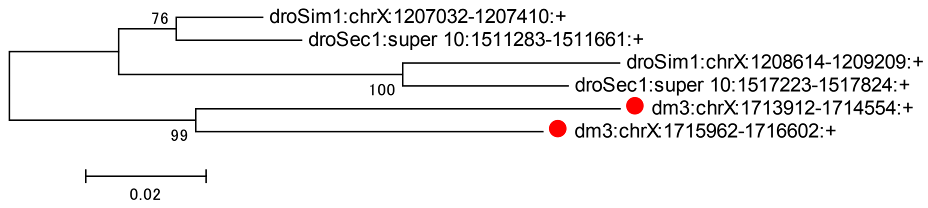

Figure S2 — Pre-speciation block 30

A) Duplication block in melanogaster subgroup (DNA sequence, Kimura's distance)

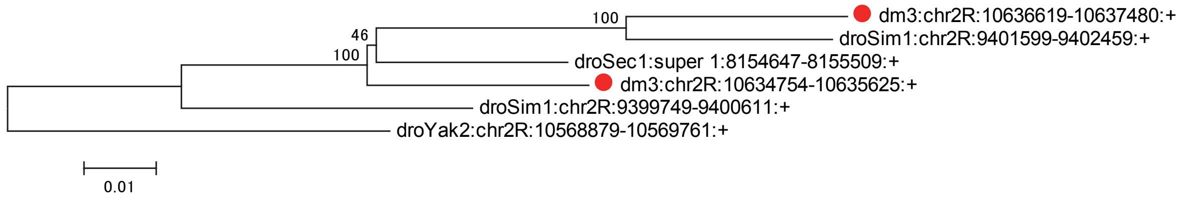

B) Window plot

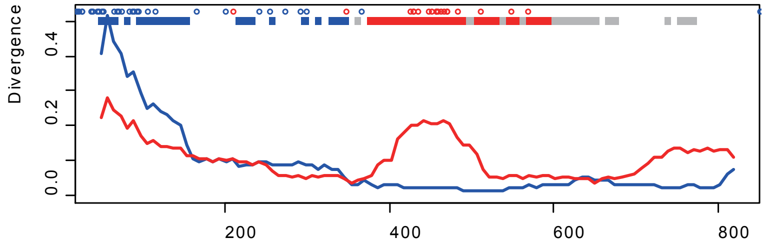

Supplement: Figure S2 — (A) Phylogenetic analysis of pre-speciation duplicated blocks. NJ trees of the orthologs in the D. melanogaster subgroup using the entire DNA sequences of duplicated blocks are shown. (B) Window analysis of the spatial distribution of the tree structure and orthlogous and paralogous divergences. The window size is 100 bp. The regions with the tree shape in Figure 1B (no evidence for gene conversion) are represented by blue bars at the top of the panel, and those with the tree shape in Figure 1C (evidence for gene conversion in both the two species) are represented by red bars. Gray bars represent the regions with other tree shapes including the one in Figure 1D, and the regions with no outgroup data (i.e., D. yakuba and D. erecta) are shown in blank. The positions of type-N and type-C sites are presented by blue and red circles, respectively. The distribution of the divergence between the paralogs in D. melanogaster is shown by the blue curve, while that of the orthologous divergence between D. melanogaster and D. simulans is shown by the red curve. Window analysis was not applied to Pre1 and Pre29 because of the lack of data of the D. yakuba data. (5.46 MB PDF) [file pgen.1000305.s002.pdf]
