# Supplementary figures and images for: Duplication and Gene Conversion in the Drosophila melanogaster Genome
Source: PLoS Genet. 2008 Dec 12;4(12):e1000305. doi: 10.1371/journal.pgen.1000305 (PMC2588116; doi:10.1371/journal.pgen.1000305)

Figure S3 — Protein trees for Pre28

A Peak 1 (181 - 267 aa)

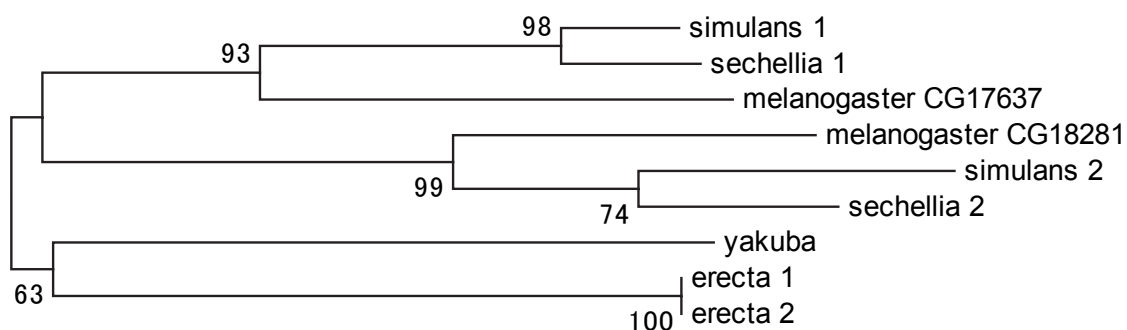

B Peak 2 (364 - 391 aa)

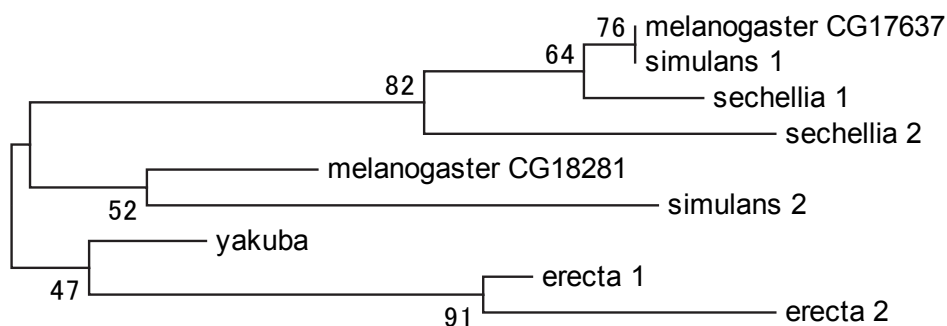

C Other regions

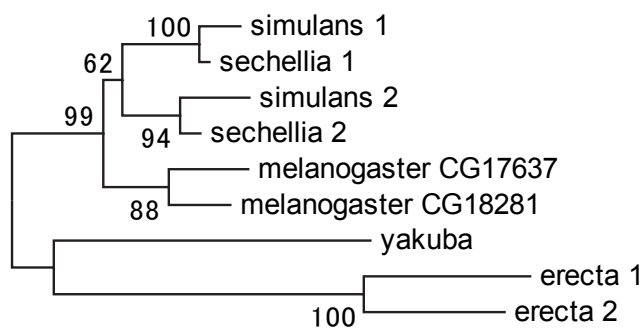

0.05

Supplement: Figure S3 — The distributions of codon adaptation index (CAI, Sharp and Li 1987 Nucleic Acids Res. 15, 1281–1295) for single-copy genes (open circles) and for our duplicates (bar graph). (0.06 MB PDF) [file pgen.1000305.s003.pdf]
